# A bioinspired neural architecture search based convolutional neural network for breast cancer detection using histopathology images

**DOI:** 10.1038/s41598-021-98978-7

**Published:** 2021-10-07

**Authors:** Olaide N. Oyelade, Absalom E. Ezugwu

**Affiliations:** School of Mathematics, Statistics, and Computer Science, University of KwaZulu-Natal, King Edward Avenue, Pietermaritzburg Campus, Pietermaritzburg, KwaZulu-Natal 3201 South Africa

**Keywords:** Cancer, Computational biology and bioinformatics, Mathematics and computing

## Abstract

The design of neural architecture to address the challenge of detecting abnormalities in histopathology images can leverage the gains made in the field of neural architecture search (NAS). The NAS model consists of a search space, search strategy and evaluation strategy. The approach supports the automation of deep learning (DL) based networks such as convolutional neural networks (CNN). Automating the process of CNN architecture engineering using this approach allows for finding the best performing network for learning classification problems in specific domains and datasets. However, the engineering process of NAS is often limited by the potential solutions in search space and the search strategy. This problem often narrows the possibility of obtaining best performing networks for challenging tasks such as the classification of breast cancer in digital histopathological samples. This study proposes a NAS model with a novel search space initialization algorithm and a new search strategy. We designed a block-based stochastic categorical-to-binary (BSCB) algorithm for generating potential CNN solutions into the search space. Also, we applied and investigated the performance of a new bioinspired optimization algorithm, namely the Ebola optimization search algorithm (EOSA), for the search strategy. The evaluation strategy was achieved through computation of loss function, architectural latency and accuracy. The results obtained using images from the BACH and BreakHis databases showed that our approach obtained best performing architectures with the top-5 of the architectures yielding a significant detection rate. The top-1 CNN architecture demonstrated a state-of-the-art performance of base on classification accuracy. The NAS strategy applied in this study and the resulting candidate architecture provides researchers with the most appropriate or suitable network configuration for using digital histopathology.

## Introduction

Deep learning (DL) models represent a family of machine learning algorithms that assign the task of feature extraction and classification to the machine, thereby eliminating semi-autonomous feature extraction. Although the application of the feature extracted may not only be applied to image classification tasks, the DL models have achieved an impressive performance in image classification^1^. Nevertheless, most of the outstanding performances recorded by DL models were largely dependent on handcrafted neural networks requiring some human expertise and domain-specific knowledge. This limited the possibility of designing best-performing networks for application to new domains because amateurs would have to rely on pre-trained DL models, an approach referred to as transfer learning. In addition to that challenge, a significant effort is required to manually design deep neural network architecture as it is a laborious task, often limiting the exploration of network search spaces. The reliance on human expertise in achieving state-of-the-art architectures resulting from this manual approach is due to the use of manual backbone architectures or micro building blocks^2^. A new research field, namely neural architecture search (NAS), aimed at using reinforcement learning (RL) or optimization algorithms to automate the design of DL architecture, has been proposed^3^. The NAS technique allows for the design of high-performing models by using search strategy based on RL or optimization algorithms to search and design neural architectures iteratively. Initial candidate solutions (neural architectures) are generated based on a constrained formal definition of a search space allowing the search strategy to apply an evaluation function in realigning the networks during iteration. A search space, search strategy, and evaluation function are the three components of a NAS model that allows for automating neural architecture engineering. Studies have shown that DL architectures, convolutional neural networks (CNN) specifically engineered using these approaches, have outperformed the handcrafted architectures applied to some problems^4–6^. The NAS methods allow for obtaining the best performing CNN design suitable for a classification or learning problem for which the model is trained.

There is an overlap between the application of optimization algorithms in the tuning of hyperparameters and the use of optimization algorithms in automating neural architecture design. The former, referred to as hyperparameter optimization, aims to tune the hyperparameter of the already designed neural network. In contrast, the latter describes NAS, which embodies search space, search strategy and evaluation operations. In building search space for NAS, sequential layer-wise, cell-based, hierarchical structure, and memory-bank representations have been applied in literature^7^. These representations generated a population of networks that NAS-based search (optimization) algorithms sample to obtain the best performing network. The most frequently used algorithms for the search process in NAS are random search (RS), reinforcement learning (RL), evolutionary algorithms (EA), progressive decision process (PDP), and gradient descent (GD). The EA approach supports neural network topologies' evolvement using an algorithm such as the genetic algorithm (GA). The EA approach mirrors the novel Ebola virus disease propagation optimization model proposed in this study. To reward the outcome of the search algorithm, evaluation strategies are employed as feedback to the search algorithm for improving its task of outputting high-performance candidate architecture. Strategies such as the full training of all candidate solutions (networks) from scratch; training with a smaller dataset with fewer iterations (proxy evaluation); weight sharing among semblance networks; and one-shot architecture with sharing of weight parameters^8^ have been widely used for evaluating the performance of potential solutions.

On the other hand, digital histopathology images are digitized images curated from the examination of biopsy samples on a microscopic slide for detecting cancer growth, a process known as histopathology. These digital histopathology images present a difficult deep learning problem compared to digital mammograms images^9^. The latter category of images often captures a case-based representation using an image sample, while in the former, a patient case is represented by large sets of images resulting from different observations of biopsy situations. Additionally, a list of subtle signs of malignancy is required to be checked to rule out benign cases in histopathology images. For instance, detecting the presence of disruption of basement membranes, marked cellular atypia, metastasize, and mitosis is an important indicator of breast cancer in histopathology images. Moreover, pathologists are expected to apply their years of experience to observe these images, classifying them as normal tissue, benign tissue, in situ carcinoma, and invasive carcinoma. Classification of these tissues often presents a complex task owning to background structures and heterogeneity in such images^10^. However, a gold standard for detecting breast cancer is the use of histopathology images above mammography images^11^. Ensuring the use of this standard will help improve the detection of breast cancer which accounts for about 32% of all cancer cases^12^. Finding an optimal neural architecture for this learning problem often proves difficult and daunting even for those with expertise in neural network design. The application of the NAS approach in designing the best performing neural architecture for this task remains promising.

However, finding candidate neural architecture to address the learning process that uses histopathology images and generates an efficient potential search space is extremely challenging. It is argued that the efficiency of a search space determines the quality of neural architectures that a NAS model can output^13^. Also, the limitations often placed on the size of this search space have mostly inhibited the upper bound of the optimal neural architectures^14^. This echoes the concern of Garg et al. ^2^ which noted that current NAS methods depend heavily on manual efforts in the search space design and are still being prototyped after the approach used in building models before the advent of the NAS^2^. In addition, manually tweaking network configuration and hyperparameters is time-consuming and challenging. Applying an optimization algorithm to fine-tune hyperparameters alone might not be sufficient, hence the need to improve the search strategy of NAS by designing a complete CNN architecture using the enhanced NAS model. Besides these issues of search space and search strategy and the viability of NAS notwithstanding, we found no study investigating the use of NAS models to the task of breast cancer histopathology dataset except those using a manual approach^15–26^. Considering the great benefit the neural network architecture holds in detecting and staging breast cancer using histopathology images^27^, we seek to address the research question: Is it possible to generate new state-of-the-art CNN architecture using NAS model, driven by biology-based optimization strategy, for solving classification problem on histopathology images?

This study proposes a new NAS model to generate candidate CNN architecture for detecting breast cancer using histopathology images to address the aforementioned problems. We designed a novel block-based stochastic categorical-to-binary (BSCB) algorithm for generating and encoding CNN architectures in the search space. Also, we investigated the performance of our recently proposed optimization algorithm^28^, namely, the Ebola optimization search algorithm (EOSA) for the search strategy as compared to other existing metaheuristic optimization approaches. The study's novelty involves designing a new NAS model, the BSCB algorithm, and the enhancement of EOSA to support the formalization of solutions as CNN architectures. Secondly, this paper represents the first study to have applied the NAS model to the complex problem of classifying digital histopathology images for the detection of breast cancer. Moreover, the study aims to obtain the best performing CNN architecture to improve classification accuracy and reduce breast cancer false-positive rates in digital histopathology images.

The main contributions of this study are elaborated as follows:

i. Propose a new block-based stochastic categorical-to-binary (BSCB) algorithm for generating initial solutions and an encoding scheme for formalizing the solutions as neural networks.
ii. Propose the application of a novel bio-inspired metaheuristic algorithm (EOSA) to perform the task of adaptive search strategy for best performing neural architectures.
iii. Implement efficient image preprocessing methods using a normalization algorithm before applying the images for training.
iv. Evaluate the proposed approach by performing a wide range of extensive experiments and comparisons with existing state-of-the-art CNN architectures and other relevant recent studies dealing with the detection of breast cancer using histopathology images.

The remaining sections of the paper are organized as follows: Sect. 2 presents an overview of the Ebola Optimization Search Algorithm (EOSA) and related studies in NAS; Sect. 3 presents the methodology applied in this study; Sect. 4 presents the parameter configuration and datasets for the experimentation; Sect. 5 presents the results obtained and discussion on findings; and Sect. 6 presents the conclusion on the relevance of the study.

## Overview of EOSA and review of related studies

This section presents an overview of the optimization algorithm proposed for the NAS search strategy phase as applied in this study. The mathematical model and a summary of the Ebola optimization search algorithm (EOSA) procedure are provided in this section to conceptualise its initialization, exploitation, and exploration mechanisms. In addition, we present a review of studies focused on automation of neural network architecture design, emphasising those approaches aimed at image classification.

### Mathematical model of EOSA

Oyelade and Ezugwu proposed a novel nature-inspired metaheuristic algorithm called Ebola Optimization Search Algorithm (EOSA) which is based on the propagation model of Ebola virus disease^28^. The formalization of the EOSA algorithm is achieved in the following procedure:

1. Initialize all vector and scalar quantities which are individuals and parameters. Individuals in the sets: Susceptible (S), Infected (I), Recovered (R), Dead (D), Vaccinated (V), Hospitalized (H), and Quarantine (Q) with their initial values.
2. Randomly generate the index case (I_1_) from susceptible individuals.
3. Set the index case as the global best and current best, and compute the fitness value of the index case.
4. While the number of iterations is not exhausted and there exists at least an infected individual, then
  - For each susceptible individual, generate and update their position based on their displacement. Note that the further an infected case is displaced, the more the infection number, so short displacement describes exploitation, otherwise exploration.
    i. Generate newly infected individuals (nI) base on (a).
    ii. Add the newly generated cases to I
  - Compute the number of individuals to be added to H, D, R, B, V, and Q using their respective rates based on the size of I
  - Update S and I base on nI.
  - Select the current best from I and compare it with the global best.
  - If the condition for termination is not satisfied, go back to step 6.
5. Return global best solution and all solutions.

The mathematical model of the procedure above follows: update of Susceptible (S), Infected (I), Hospitalized (H), Exposed (E), Vaccinated (V), Recovered (R), Funeral (F), Quarantine (Q), and Dead (D) is governed by a system of ordinary differential equations derived based on those in^29,30^. Differential calculus is a branch of calculus which in turn is a branch in mathematics. The former deals with the rate of change of one quantity with respect to another, while the latter deals with finding different properties of integrals and derivatives. In our case, the application of differential calculus intends to obtain the rates of change of quantities S, I, H, R, V D, and Q with respect to time ***t***. Hence, the Eqs. (1, 2, 3, 4, 5, 6, and 7) for S, I, H, R, V D, and Q respectively as follows:

1 $$\frac{\partial S(t)}{\partial t}=\pi -\left({\upbeta }_{1}\mathrm{I}+ {\upbeta }_{3}\mathrm{D}+ {\upbeta }_{4}\mathrm{R}+ {\upbeta }_{2}\left(\mathrm{PE}\right){\eta}\right)S-({\tau S}+{\Gamma I})$$

2 $$\frac{\partial I(t)}{\partial t}=\left({\upbeta }_{1}\mathrm{I}+ {\upbeta }_{3}\mathrm{D}+ {\upbeta }_{4}\mathrm{R}+ {\upbeta }_{2}\left(\mathrm{PE}\right)\uplambda \right)S-\left(\Gamma +\upgamma \right)I -\left(\uptau \right)\mathrm{S}$$

3 $$\frac{\partial H(t)}{\partial t}=\mathrm{\alpha I}-(\upgamma +\mathrm{\varpi })\mathrm{H}$$

4 $$\frac{\partial R(t)}{\partial t}=\upgamma I-\Gamma R$$

5 $$\frac{\partial V(t)}{\partial t}=\upgamma I -(\upmu +\mathrm{\vartheta })V$$

6 $$\frac{\partial D(t)}{\partial t}=\left(\mathrm{\tau S }+\mathrm{\Gamma I}\right)-\updelta D$$

7 $$\frac{\partial Q(t)}{\partial t}=\left(\mathrm{\pi I}-(\mathrm{\gamma R}+\mathrm{\Gamma D}\right))-\upxi Q$$

Table 1 presents the definition of the parameters and symbols used in the EOSA model design for the proposed bio-inspired neural architecture search.

**Table 1:** A description of notation and coefficients used in Eqs. (1)–(7).

Symbols	Descriptions
π	Recruitment rate of susceptible human individuals
Ŋ	Decay rate of Ebola virus in the environment
Α	Rate of hospitalization of infected individuals
Γ	Disease-induced death rate of human individuals
β_1_	Contact rate of infectious human individuals
β_2_	Contact rate of pathogen individuals/environment
β_3_	Contact rate of deceased human individuals
β_4_	Contact rate of recovered human individuals
γ	Recovery rate of human individuals
τ	Natural death rate of human individuals
δ	Rate of burial of deceased human individuals
ϑ	Rate of vaccination of individuals
ϖ	Rate of response to hospital treatment
μ	Rate of response to vaccination
Ξ	Rate of quarantine of infected individuals

In Table 1, we summarise the notations or coefficients used in Eqs. (1)–(7). This study proposes to adapt the EOSA algorithm to searching neural network architectures for improved classification tasks. Meanwhile, we review related studies to investigate the approach adopted for the optimization stage of the search strategy of recent NAS models. The following sub-section reveals our findings from this review.

### Review of related studies on neural architecture search

Neural architecture search (NAS) models consist of search space, search strategy and evaluation strategy. Several studies in the literature have demonstrated variant techniques for formulating each of the components of NAS. In this section, we present the review in chronological order to understand research trends in the field.

Cortes et al.^31^ proposed a NAS framework, namely AdaNet, which applied adaptive structural learning technique for the search strategy. The learning strategy utilized a data-dependent generalization method which successfully learnt both the structure of the network and its weights automatically to yield an optimal network structure. In a related study, Negrinho and Gordon^32^ applied modular language techniques for the design of the search space for their NAS framework. The technique allows for populating the complex search spaces with representations of CNN architectures and their hyperparameters. Experimentation was done using three search algorithms, namely random search, Monte Carlo tree search (MCTS), and sequential model-based optimization (SMBO) over the search space. Garg et al.^2^ proposed an approach called ReNAS, which represents the search space for architectures as a direct acyclic graph (DAG), which consists of basic operations. The resulting graph was then mapped to a neural network to search for candidate solutions using a differentiable architecture search approach. The results obtained in the study showed that although the approach outperformed handcrafted architectures, itcould not, however, achieve superiority over state-of-the-art NAS methods but was competitive in performance.

Wang et al.^33^ addressed the shortcomings of using only the Hyperband algorithm for searching optimal neural architecture. As a result, the Hyperband algorithm and Bayesian optimization technique were hybridized to design the search strategy of NAS. The hybridization aims to build a memory to recall previous configurations when sampling the next trial configuration in searching for the optimal CNN configuration. In another study focused on improving the NAS search strategy, Huang et al.^34^ proposed using a greedy technique to enhance the neural architecture search. The resulting GNAS framework was applied to address the problem of finding optimal CNN architecture for extracting features from images by exploiting an efficient greedy search approach. The greedy technique achieves its search strategy by splitting a bigger neural architecture into smaller versions optimized in a stepwise manner. The GNAS automatically discovered optimal tree-like CNN architecture for learning and extraction of multi-attribute. Using another approach for search strategy, Weng et al.^35^ applied differential architecture (DARTs) search method to design CNN architecture. The resulting search strategy was built into a convolutional neural search architecture (CNAS) framework. The proposed DART-based search strategy finds architectures from a search space utilizing both shuffle operation and squeeze-and-excitation. We, however, found their approach to be sub-optimal when compared with the use of evolutionary-based optimization techniques, as seen in the works of Erivaldo et al.^36^ and Liu et al.^37^. The two studies approached the design of search strategy of their NAS framework using particle swarm optimization (PSO) and Genetic Algorithm (GA)-based, respectively. The optimization mechanism of PSO was employed for a direct encoding strategy. In contrast, the optimization task of GA was supported by an experience-based greedy exploration strategy and transfer learning techniques.

Considering the cardinal role of the search strategy in the NAS model, we reviewed recent studies to observe the approaches applied. For instance, Krishna et al.^38^ proposed two techniques, namely reinforcement learning strategy and attention-based mechanism with simplified transformer block method for the search strategy of NAS and improving hyperparameter candidate neural architecture, respectively. The study uses a two-stream attention-based mechanism to model hyper-parameter dependencies and a simplified transformer block. In contrast, the second method aimed to remove layer normalization, the former models the policy network for searching the space. The authors reported that the performance of their method surpasses most methods in NAS-Bench-101 benchmarked models.

A similarity to the works of Erivaldo et al.^36^ and Liu et al*.*^37^ is seen in the study by Calisto and Lai-Yuen^39^. This study applied evolutionary algorithms to search for neural networks in the search space to discover high-performing and efficient neural architecture. The optimization algorithm is rewarded if it can discover architectures with improved classification accuracy and reduced hyperparameters size. The resulting architecture which comprises a self-adaptive 2D and 3D ensemble of FCNs used for 3D medical image segmentation was code-named AdaEn-Net. In a similar approach, Chen and Li^40^ proposed using an evolutionary algorithm for the search strategy of a search space. The search space is composed of a major super-network whose weight is shared with sub-network architectures in obtaining an optimal candidate network. This optimal architecture is derived from a collection of most performing or excellent architectures by examining the commonalities of these architectures^40^.

Wang et al.^41^ proposed what is referred to as DC-NAS, which was derived from a divide-and-conquer (DC) approach to the NAS problem. The study applied the DC method to obtain the best performing sub-graphs of potential complete network architecture. Meanwhile, sub-graphs are first clustered based on their similarity so that best performing sub-graphs in a cluster are merged with other optimal sub-graphs in related clusters to form a new architecture. The resulting optimal sub-graphs combine to form a new neural architecture that is effective and efficient. On the issue of NAS search space, we found the work of Cassimon et al*.*^42^ which uses a cell-based representation approach for search space very interesting. The method adapted reinforcement learning to optimise cells for detecting the two types of networks, namely Recurrent Neural Network (RNN) and Convolutional Neural Network (CNN). The study considered a network optimal if it could successfully predict spoken words and classify RGB images on an embedded device. The study's main contribution is the proposal of an efficient neural architecture search (ENAS) fitted for embedded devices and with improving performance network architecture. In a similar approach, though using a different NAS-based method, Fan et al.^43^ jointly searched for operations like LSTM and CNN from a search space using gradient-based neural architecture optimization (NAO) technique. The search space combined two heterogeneous spaces with network operations space and dropout space. The former consists of basic network operations, while the latter space consists of dropout rates. The resulting networks are those whose architectures and hyperparameters are well optimized for neural machine translation (NMT).

An improvement to the work of Cortes et al.^31^ is reported in the study of Dai et al*.*^44^. The authors employed a NAS framework driven by the use of the AdaNet technique. The focus of the improvement is achieving a better search space and search strategies for obtaining the optimal structure of the CNN architecture and optimising the weights of the CNN architecture. AdaNet utilizes a simple linear model representing the search space and then gradually augments the network with more neurons and additional layers until an optimal network architecture is obtained. Each step in building the resulting architecture requires that a Gradient-decent-based optimization method using Momentum be applied. The study's outcome is a CNN architecture used for three classes (3-hinge gyral vs 2-hinge gyral vs sulcal) classification in f-MRI signals classification problem. In a different approach, Gheshlaghi et al*.*^45^ proposed a NAS model by applying the binary gate method to search strategy through stacking of cells upon cells of sub-networks using primitive operations. These cells consist of Down-Sampling Cell (DownSC) and Up-Sampling Cell (UpSC) whose designs are automated into the NAS process. The resulting optimal neural network architecture is expected to outperform handcrafted architecture, which is purposed for the same task of retinal layer segmentation in Optical Coherence Tomography (OCT) scans.

Chen et al.^46^ proposed a single-stage NAS framework named you only search once (YOSO) for automating the process of finding the optimal deep neural network (DNN), which are used for co-designing of software/hardware. The need for co-design of software and hardware by the resulting DNN further swelled the volume of the search space with hyperparameters of the DNN and hardware design parameters. The study applied reinforcement learning with LSTM for the search strategy. The resulting NAS framework applied a multi-objective reward system aimed at maximizing accuracy, power, and QoS. Meanwhile, several DNNs are generated from basic operations to formulate the search space. An interesting aspect of the study is the use of an auxiliary HyperNet that voids the training of candidate DNNs before applying resulting weights for evaluating their performances in terms of accuracy. In another study, Guo et al.^47^*,* proposed a variant of NAS capable of generating neural architectures using an inference model. The neural architecture generating (NAG) model learns from a Pareto frontier, which guides optimal architectures based on the given budget for the target system on which the resulting architecture is expected to be used. On the other hand, Zhang et al.^48^ addressed the problem of the non-convexity of NAS through the use of an adaptive, scalable neural architecture search method (AS-NAS). The scalability of AS-NAS was achieved through a search strategy that combined a simple reinforcement learning, namely: reinforced I-Ching divination evolutionary algorithm (IDEA) and variable-architecture encoding strategy.

In a similar approach to Krishna et al.^38^ and Weng et al.^35^, though an improvement on the approach, He et al*.*^49^ proposed a special kind of NAS model called attention-guided differentiable architecture search (A-DARTS), which adopts a mechanism for reducing the sensitivity of initialization of searched space. Also, Xu et al*.*^50^ improved the efficiency and stability of searched networks using the Partially-Connected DARTS (PC-DARTS) approach. The PC-DARTS improves the search strategy by randomly selecting a small subset of channels for partial channel connection to overcome over-fitting the search networks.

Several studies have proposed new variants of the NAS model. For instance, Ru et al.^51^, applied the technique of Bayesian optimization (BO) to the design of the NAS model to obtain a new model known as interpretable neural architecture search (INAS). The proposed INAS uses graph-like search spaces while combining the Weisfeiler-Lehman graph kernel with a Gaussian process surrogate with BO for the search strategy. Fu et al*.*^52^, addressed the problem of incremental learning in the classification of images through the approach of neural architecture search for incremental learning *(*NASIL). This was done by using reinforcement learning, parameter-sharing mechanism, and Long Short-Term Memory (LSTM). Also, Lin et al.^53^ added novelty to the approach of NAS by improving the evaluation strategy, which replaces an accuracy predictor with zero-shot in the ranking of searched architectures. The resulting value from the zero-shot operation is maximized using an inference budgets model called Zen-NAS.

On the other hand, Liu et al*.*^54^*,* applied an evolutionary method to optimise weight-sharing parameters when searching for optimal neural architectures. This search strategy, called continuous particle swarm optimization (CPSO-Net), computes the gradient of networks resulting from shared parameters of candidate operations to obtain candidate architecture. Lastly, Liang et al.^55^*,* applied a variant of NAS to generate optimal feature pyramid networks (FPNs). The resulting One-Shot Path Aggregation Network Architecture Search (OPANAS) approach uses a one-short strategy for searching for candidate FPNs that are drawn from DAG-based FPNs search space.

The review presented in this section, and summarized in Table 2, demonstrates that different methods have been applied to improve the components of the NAS model. The components which have received more research attention are the search space encoding strategy and the search strategy. Our findings revealed that most of the studies had applied reinforcement learning techniques, evolutionary and use of metaheuristic algorithms. We discovered that the most promising approach is seen in studies that used evolutionary or computational biology methods for search strategy. Hence, this study aims to improve the NAS search strategy by using EOSA, a bio-inspired optimization algorithm, to generate optimal neural architecture in classifying histopathological images to detect breast cancer. In addition, a novel search space encoding algorithm is proposed to allow for good coverage of the potential CNN architectures. The following section details the search space and search strategy proposed in this paper.

**Table 2:** Summary of the reviewed studies.

References	Search space	NAS search and optimization method	Evaluation strategy
Cortes et al.^31^	Simple network grown incrementally	Adaptive structural learning (AdaNet)	Binary classification accuracy
Negrinho and Gordon^32^	Tree-structured search space	MCTS and SMBO	Training and validation
Wang et al.^33^	AlexNet and LeNet hyperparameters	Hyperband algorithm and Bayesian optimization	Classification accuracy
Huang et al.^34^	Global architecture	Greedy search approach	Mean prediction accuracy
Weng et al.^35^	Primitive operations and intermediate nodes	DARTs	Measuring loss and accuracy
Erivaldo et al.^36^	Random CNN architectures initialization	PSO search strategy	Crossentropy loss and velocity computation
Liu et al.^37^	Residual blocks	GA search strategy	Fitness function for image quality measurement
Garg et al.^2^	Hierarchical structure using DAG	Differentiable architecture search	Surrogate approach
Krishna et al.^38^	NASBench-101 search	Reinforcement learning	Actor-critic algorithms
Calisto and Yeun^39^	Basic operations and corresponding hyperparameters	Evolutionary algorithm	Classification accuracy and hyperparameter reduction
Wang et al.^41^	Cell-based representation	Divide-and-conquer (DC) approach	k-means base clustered evaluation
Cassimon et al.^42^	Cell-based representation	Reinforcement learning	Multi-objective evaluation
Fan et al.^43^	Hybrids of cell-based representation	Gradient-decent-based neural architecture optimization (NAO)	Minimization of regression and reconstruction losses, and dropout rates
Dai et al.^44^	AdaNet: Hierarchical structure	Gradient-decent-based using Momentum	Maximizing classification accuracy
Gheshlaghi et al.^45^	Cell-based representation of primitive operations	Gradient-based approach for binary gate method	Training from scratch
Chen et al.^46^	Basic operations	Reinforcement learning using LSTM	HyperNet based accuracy evaluator and hardware performance predictor
Chen and Li^40^	Weight sharing strategy from a major super-network	Evolution algorithm method	Commonalities among best performing architectures
Guo et al.^47^	Basic operations	Inference model learning from Pareto frontier parameters	Model performance and computational cost
Zhang et al.^48^	Basic operations	Reinforcement learning and evolutionary algorithm	Minimization of loss function
Hu et al.^49^		Attention-guided differentiable mechanism	Classification accuracy
Xu et al.^50^	Super-network	Partially connected DARTS	Error rates of searched networks
Ru et al.^51^	Graph-like search spaces	Bayesian optimization	Performance evaluation of motifs
Fu et al.^52^	Basic operations	Reinforcement learning and LSTM	Quantified-parameter evaluation mechanism
Lin et al.^53^	A single randomly initialized network	Inference budgets model	Zero-shot approach
Liu et al.^54^	A SuperNet	Particle swarm optimization	
Liang et al.^55^	DAG-based FPNs	One-short search strategy	Detection accuracy

## Methods

This section is focused on the design methods of the three (3) components of the neural architecture search (NAS). First, we present our proposed NAS model which demonstrates the interoperability of the 3 components. Secondly, the design of a novel search space encoding algorithm that defines a population of initial CNN solutions is discussed. Thirdly, the neural search strategy which is based on the Ebola optimization search algorithm (EOSA) method, is presented. Fourthly, we demonstrate how the multi-objective evaluation strategy is computed and how its results are passed back to the search strategy for refinement purposes.

### The neural architecture search (NAS) model

This sub-section gives a high-level overview of the proposed NAS model, which shows basic operations for the search space, the search strategy, and the performance evaluation strategy. The overall aim of the model is to guide the selection process of best performance arbitrary CNN architecture, from the search space, for solving the classification problem.

**Figure 1:**
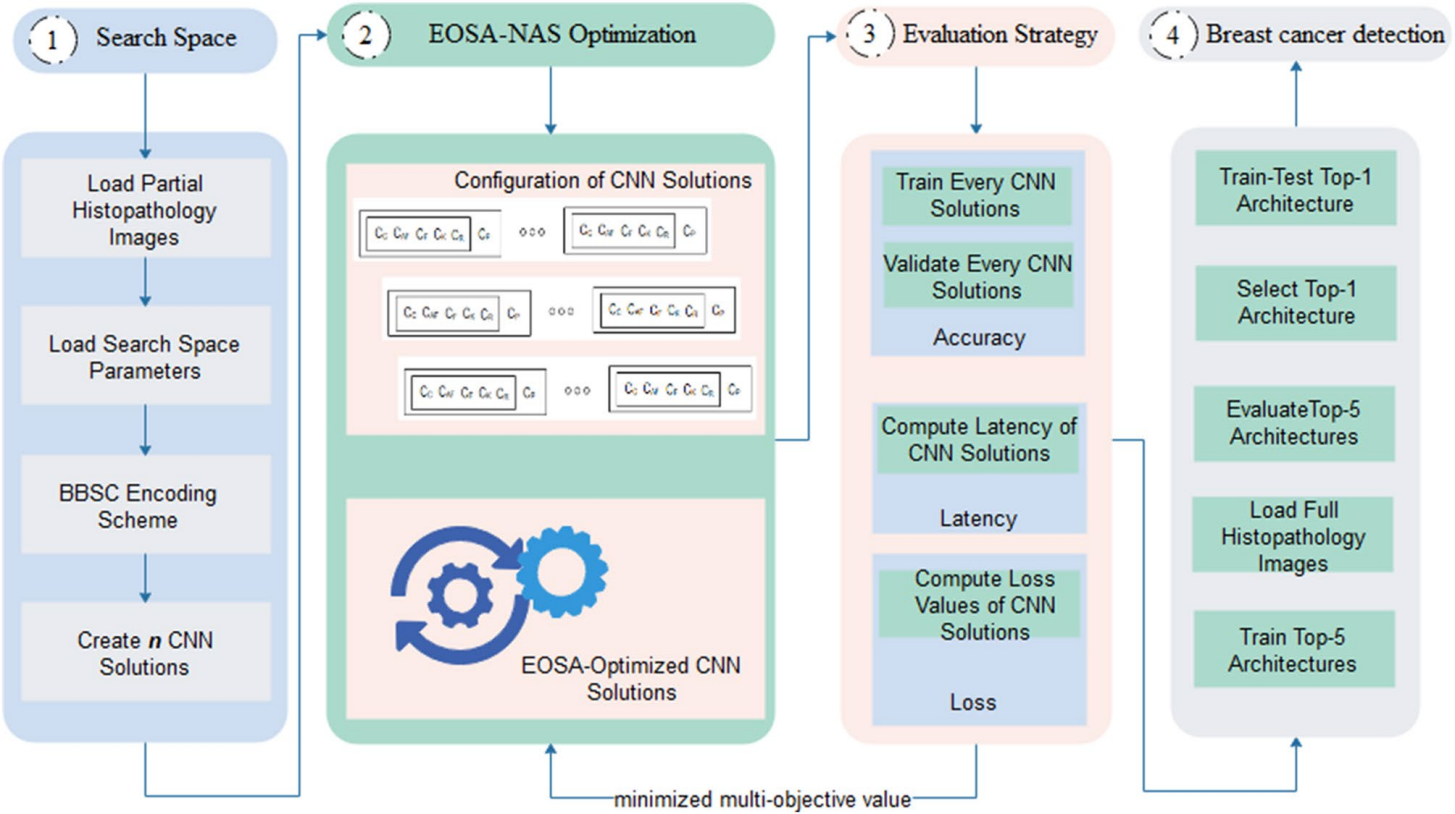
The proposed EOSA-NAS model consisting of four components: the search space, EOSA-NAS search strategy, evaluation strategy and the breast cancer detection module using the top-5 and top-1 CNN architectures.

The proposed NAS model is presented in Fig. 1 and shows the three major components of NAS. In addition, a mechanism for evaluating the best performing architecture resulting from the search strategy is also provided. The following is a brief discussion of each component:

1. **Search space** The proposed model shows how an encoding scheme is used to generate ***n*** potential initial solutions, representing CNN architectures. The proposed encoding scheme aims to create a pool of potential configurations of basic operations and hyperparameters of n CNN architectures that are capable of yielding the best performance.
2. **Search strategy** A bioinspired EOSA-based search strategy iteratively optimizes each CNN solution from a pool of potential solutions located in the search space. For each iteration, the configuration of each CNN solution is improved towards learning the classification problem. This is attained in conjunction with a mechanism for evaluating performance based on high accuracy, reduced loss value, and low latency.
3. **Evaluation strategy** We designed a mechanism for measuring and estimating the performance of CNN models resulting from the optimization operations on the search space. This allows for passing a kind of reward to the search algorithm to support the process of finding a candidate CNN solution. To minimize the computational cost of the evaluation, we trained the CNN models for few epochs and computed the result of the objective functions, namely classification accuracy, loss function, and CNN architectural latency.
4. **Evaluation of top-performing CNN models** After an exhaustive optimization process, the top-5 performing CNN models are chosen from the ***n*** solutions. These top-5 are subjected to further comparative analysis to measure and discover their capability in solving the classification problem—the problem of detecting the presence of breast cancer in histopathological images. Here, the top-5 CNN architectures are subjected to full training using the complete datasets.

The following sections present detail on the design and applicability of each component in the NAS model illustrated by Fig. 1.

### The search space and encoding scheme

The quality of a search space determines the performance of both the initial and candidate CNN solutions in NAS models. Also, the encoding scheme applied to a search space directly impacts the complexity of the neural search strategy. Hence, it is necessary to carefully choose the technique to represent a search space in a NAS model. In this section, we present the design of a novel search space encoding initialization and encoding scheme. The design is based on a block-based approach. Firstly, the proposed encoding strategy is designed to generate potential initial CNN solutions exhaustively. Secondly, the design also models each CNN solution in such a manner as to allow the evaluation of the multi-objective functions inexpensively. Thirdly, the scheme provides scalable and easy navigation within the search space using the search space algorithm. We propose a block-based stochastic categorical-to-binary (BSCB) encoding scheme that maps each unique parameter label to an integer value when constructing the search space.

The categorical feature or parameter is first converted into a numeric value using an ordinal encoder. This strategy allows for digitizing each convolutional operation and hyperparameters of the CNN solution, which then allows for the efficient representation in the solution space. Once the categorical transformation is achieved, we binarize the resulting integer values. Each binarized value is then bounded within its lower and upper bounds to ensure that it represents a valid CNN architecture. The encoded parameters are then used for building a multi-block-based schematic representation of a CNN model. The resulting blocks are stacked in an ordinal fashion based on the traditional approach of designing CNN architectures. A well-stacked group of blocks represents a potential CNN solution generated into the search space. The implication of this is that CNN architectures are designed on the fly with no prior handcrafted configurations.

To achieve the encoding scheme described in the previous paragraph, we provide a list of potential parameters from which blocks are encoded. These parameters, listed in Table 3, represent the convolutional operations and hyperparameters of a vanilla CNN model. The listing allows for a wider range of combinations of values for each parameter. This outcome is a pool of rich potential initial CNN solutions for use by the search strategy.

**Table 3:** Categorization of parameters based on the block encoding scheme for representation of the hyperparameters of convolutional neural network.

(Min, max) no. of blocks in BSCBE	Block category	CNN Hyperparameters	Notational Representation	Lower Bound	Upper Bound
(1, 1)	General hyperparameter block	Batch size/mode	G_b_	0	2
		Learning rate	G_α_	0	8
		Optimization algorithm	G_o_	0	7
		Epoch	G_e_	1	2
(0,1)	Input-Zeropadding block	Whether to zero-pad inputs or not	Z_α_	0	1
(1, N)	Convolutional layer block	Number of conv-pool blocks	C_*L*_	1	6
		Number of convolutional blocks in C_l_	C_*C*_	0	2
		Choice of activation function per convolutional layer	C_*AF*_	0	2
		Number of kernel	C_*K*_	3	10
		Kernel size	C_*F*_	0	10
		Pool size	C_*PS*_	0	2
		Pool operation type	C_*PT*_	0	1
		Weight regularization operation	C_*R*_	0	2
(1, 2)	Fully connected block	Number of dense (fully-connected) layers	F_*L*_	0	1
		Activation function for the layer	F_*AF*_	0	2
		Use of dropout layer	F_*D*_	2.0	2.2
		Weight regularization operation	F_*R*_	0	2
(1, 1)	Loss function block	Choice of loss function	LF_*L*_	0	2

The list of parameters that constitutes the search space includes the batch size of samples used for input, learning rate, optimization algorithm, the number of convolutional layers, number of kernels, kernel size, the activation function of each convolutional layer, the pooling type and size, the number of dense layers, the connectivity pattern, the activation function, weight regularization techniques, and the dropout for each dense layer. To generate potential CNN solutions into the search space, the following describes how the proposed block-based encoding scheme utilizes these parameters as defined in Table 3.

First, we note that when required for generating an arbitrary CNN solution, each parameter is derived using Eq. (8). Moreover, an arbitrary CNN solution combines a number of these parameters to build its blocks:

8 $${P}_{(c,i),j}={lb}_{j}+rand ({ub}_{j}- {lb}_{j})$$

where P_(c,i),j_ represents the *i*^th^ parameter in the *c*^th^ category and the *j*^th^ parameter in the list of parameters (P) to be passed to the encoding algorithm. Note that for each parameter, the *ub* and *lb* represent the upper and lower bounds, respectively. A corresponding value for each parameter is computed by generating a random number, multiplying by the difference of *ub* and *lb*, and then adding it to the lower bound. Once these values for all parameters are obtained, we proceed to the block encoding for generating CNN solutions.

In Eq. (9), we show the complete encoding of a CNN solution where each block_i_ is composed by some P_(c,i),j_. This potential auto-generated CNN solution consists of blocks of different structures arranged in an ordinal pattern to reflect the traditional architecture of CNN. Furthermore, to form the search space for the neural search algorithm, several of this CNN_solution_ are generated and represented as seen in Eq. (10), where CNN_search-space_ represents a collection of CNN solutions in the search space

9 $${CNN}_{solution}=\left\{{block}_{gy}, {block}_{iz}, {block}_{cl}1, \dots {block}_{cl}n-1,{block}_{cl}n, {block}_{fc},{block}_{lf}\right\}$$

10 $${CNN}_{search-space}=\left\{{CNN}_{solutio{n}_{1}}, {CNN}_{solutio{n}_{2}}, {\dots CNN}_{solutio{n}_{n}-1}, {CNN}_{solutio{n}_{n}}\right\}$$

A predefined number of blocks to be generated for each category are defined in Table 3. The algorithm iterates over each category and digitalizes its parameter, and computes the corresponding value of the binarized parameter mapped to it so that each category translates to a block. Note that the structures of an arbitrary *block*_*i*_ in *solution*_*x*_ might not have the same parameter value as another in the same solution. This representation allows for a radial coverage of the potential solution space to allow for an effective and efficient search space.

The binarized parameter and its corresponding category are denoted by vector v_b_ such that ${v}_{b}\in {\{0, 1\}}^{n}$ as detailed in Eqs. (11) or (12).

11 $$\left\{{v}_{b}\in {\left\{0, 1\right\}}^{n} : \sum_{i=1}^{n}{v}_{i}=1\right\}$$

12 $${v}_{b}={1}_{\rho }\left(param\right)=\left\{\begin{array}{c}0,\quad x \notin \rho \\ 1,\quad x\in \rho \end{array}\right.$$

**Figure 2:**
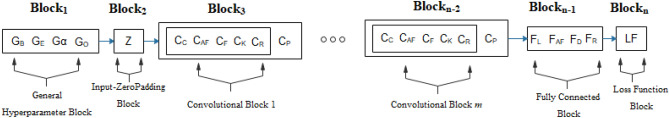
A generic representation of an encoded CNN architecture based on the parameters covered by the search space.

A general structural representation of a CNN architecture using the encoding scheme is shown in Fig. 2. While blocks 1, 2, … n-1, n form the basic structural representation for each potential CNN solution, we note that each solution could represent a more complex structure than Fig. 2. Algorithm 1 presents a pseudocode of the technique for generating all CNN solutions into the search space.
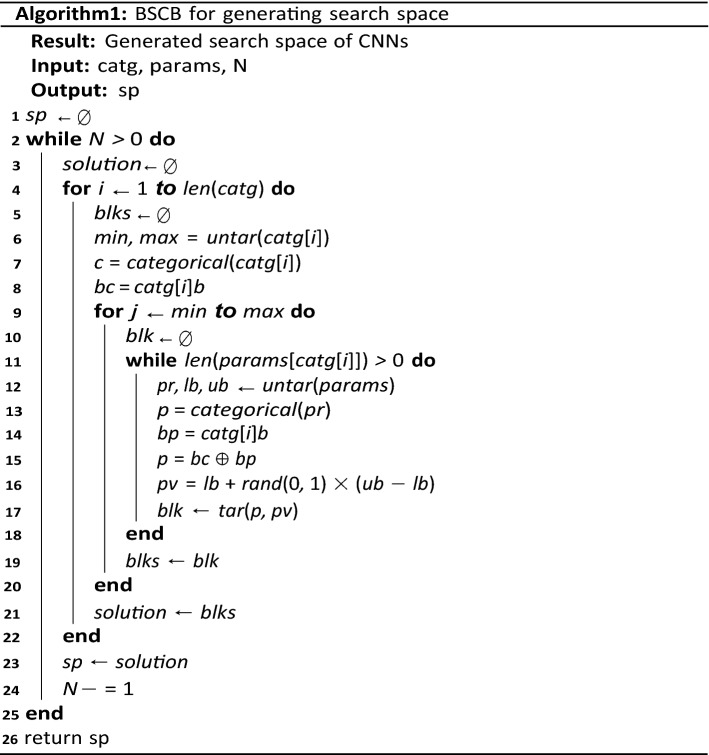

The algorithm generates ***n*** solutions for the search space by using the block-based encoding scheme. This is done by first carrying out category-based parameter extraction so that extracted parameters are then digitized. Meanwhile, the equivalent value for each parameter is computed before mapping them in a corresponding parameter-category association. Finally, blocks are formed from such mappings, which are then translated and chained into potential CNN solutions. In the next section, the application of the neural search algorithm to the search space is described in detail.

### Bioinspired EOSA search and optimization strategy

The search strategy proposed in this study is based on the Ebola optimization search algorithm (EOSA). This allows for widening the search operation in the direction of both exploration and exploitation. The outcome of the search process often yields the best performing CNN architecture for the detection and classification of breast cancer using histopathological images. The resulting CNN search algorithm is henceforth referred to as the EOSA-NAS algorithm. The EOSA-NAS algorithm explores the search space to obtain a candidate CNN architecture suitable for addressing the classification problem. The approach ensures that irrelevant candidate architectures are lined behind the potential architectures.

The search algorithm first initializes the compartments to empty sets: Susceptible (S), Exposed (E), Infected (I), Hospitalized (H), Recovered (R), Vaccinated (V), and Quarantine (Q). Thereafter, a variable is created to keep track of the top performing architectures of each iteration. The set S contains all potential solutions (CNN architectures) in the search space, ranked according to their performance based on evaluation strategy so that the most performing architectures are at the head of the queue. The CNN architecture or solution at index 0 is assigned to the exposed E set and eventually to the I set. The position of each solution is updated using (13).

13 $${mI}_{i}^{t+1}={mI}_{i}^{t}+\rho M\left({s}_{i}\right)$$

where ρ represents the scale factor of displacement such that individuals ${mI}_{i}^{t}$ and ${mI}_{i}^{t+1}$ represents the updated or current position and previous position at time t and t + 1, respectively. *M(I)* is the movement rate made by individual solutions, as shown in Eqs. (14) and (15).

14 $$M\left(I\right)=srate*rand \left(0, 1\right)+{M(Ind}_{best})$$

15 $$M\left(s\right)=lrate*rand \left(0, 1\right)+{M(Ind}_{best})$$

The search strategy is able to search within the neighborhood threshold (exploitation) using the short distance movement, *srate*. Also, the algorithm can search beyond the neighborhood threshold (exploration) using the long distance movement, *lrate*. Both *srate* and *lrate* are regulated by *neighborhood* parameters. For instance, if the computed *neighborhood* parameter is above 0.5, it is assumed the infected individual (solution) has moved beyond the *neighborhood*, hence the exploration phase. Otherwise, it is assumed it remains within the *neighborhood*, hence the exploitation phase. With this mechanism, candidate solutions or CNN architectures evolve and are placed in the I set for use in the next operation. The next operation mutates the configuration of the solutions or CNN architectures for improved performance. This mutation or optimization process is guided by the need for solutions to learn the classification problem. Every infection operation weakens the immunity of the individual (CNN architecture). The configurations of any CNN architecture in *I* are represented in Eq. (16); solutions (CNN architectures) which have recovered (*R*) have their immunity strengthened as shown in Eq. (17), and those dead individuals (*D*) are replaced by new solutions; individuals or solutions which were not infected are maintained in *S*.

16 $${NA}_{i}=cfactor * {e}^{1*l} * cos(2\pi l) + {NA}_{best}$$

17 $${NA}_{i}=rand* cfactor*({NA}_{best}- {NA}_{i})$$

where *NA* stands for neural architecture, which is the same as solutions, *cfactor* is the rate of change of the structure as determined by *neighborhood* value, and *l* is a sample drawn from a uniform distribution in the range of [−1,1]. The resulting value from the evaluation of Eqs. (16) and (17) affects the operations defined by each parameter in all blocks, as shown in Fig. 2.
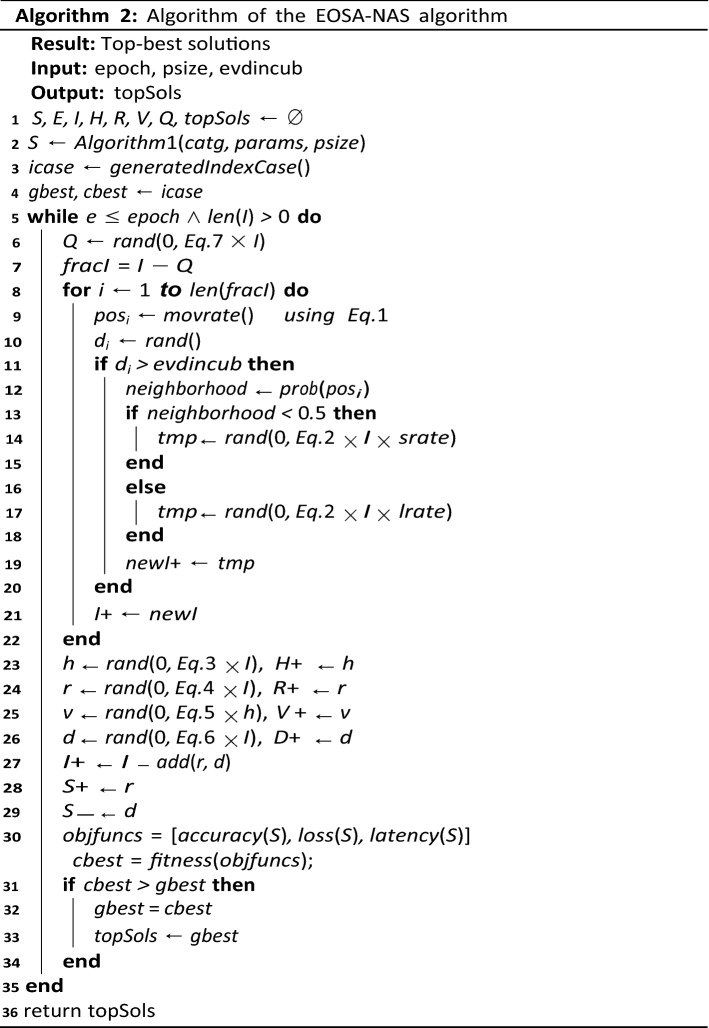


The procedure described by the mathematical model above is summarized in Algorithm 2. The use of back-arrow notation ( ←) represents storage or assignment statement, while the combined use of back-arrow and plus notations (+ ←) represents cumulative storage of values in a variable. This algorithm outlines the call to the initialization of the search space, the iteration through a given epoch for the evolvement of improved CNN architecture or solutions, and the application of the multi-objective function in obtaining the best solution. The last line returns a list of solutions representing CNN architecture with the top best at the head of the queue. The search strategy ensures that all potential architectures are evaluated based on three (3) objective functions that yield a one-value metric. The following section details this evaluation strategy.

### Evaluation strategy

The selection of the current best, at any time *t*, is computed on the set of infected individuals at that time *t*, whereas selection of the global best is based on the best performing CNN solution at the end of the training process. Performance is measured using classification accuracy on CNN training and validation, the latency of the CNN architecture, and the loss function (categorical or sparse cross-entropy). This multi-objective approach is motivated by findings from the literature that justify the need to consider factors such as model size, latency, computational time and fast response time^56^. We motivate the need for a multi-criteria evaluation strategy considering that a single-objective focused on over-classification accuracy will be inaccurate in obtaining the best performing CNN architecture. In Eqs. (18), (19) and (20) are definitions of the metrics applied for the multi-criteria evaluation strategy. Performance comparison for the similarity between CNN architectures is achieved using Eq. (21).

18 $$Accuracy=\frac{TP+TN}{(TP+TN+FP+FN)}$$

19 $$latency={time()}_{after-train}-{time()}_{before-train}$$

20 $$ loss\, function = l(w) =  - \frac{1}{N}\sum\limits_{{n = 1}}^{N} {\sum\limits_{{m = 1}}^{M} {t_{{nm}} log_{2} Pnm} }  $$

21 $$reward=\frac{1}{\left(acc+l\left(w\right)+t\right)}$$

22 $$Similarity({NA}_{i}{NA}_{j})=\left\{\begin{array}{ll}dist({NA}_{i}, {NA}_{j}),&\quad if {NA}_{i}< {NA}_{j}\\ 0, &\quad otherwise\end{array}\right.$$

where NA_i_ represents any arbitrary neural network, and the function *Similarity(NA*_*i*_*, NA*_*j*_*)* allows for comparing two neural networks in a search space.
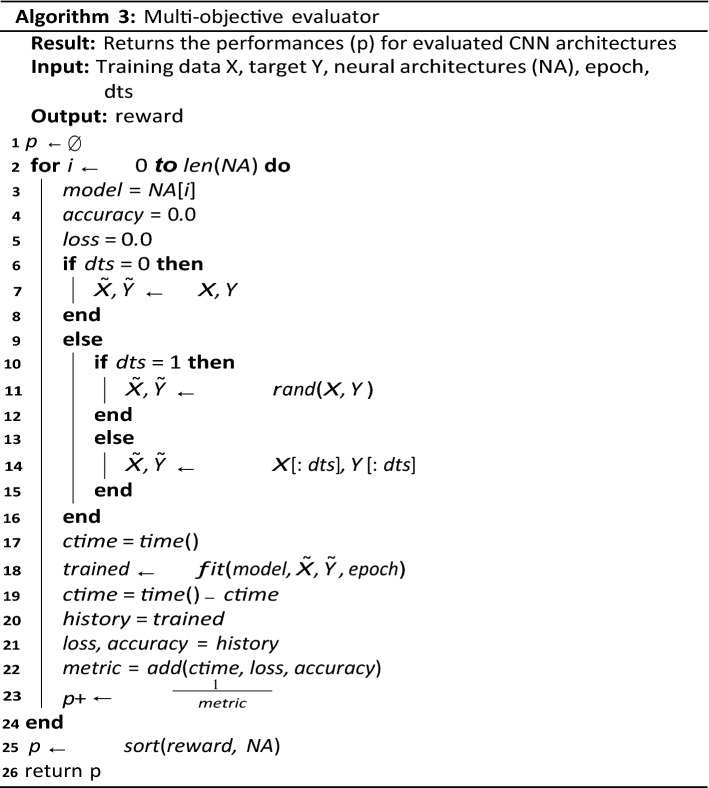


Algorithm 3 demonstrates the procedure for the evaluation of the multi-objective criteria as described previously. The expected output of the algorithm is a single value that is passed to the search strategy for improving the configuration of the CNN to achieve optimal performance. It iterates over all the CNN models and randomly generates a batch of image samples from the dataset for training the model. Once the training is completed, the training time, accuracy and loss function is computed and evaluated as a value. The configuration of Algorithm 2 to seamlessly use Algorithm 1 and 3 is presented in the next section, which is focused on the experimentation of the proposed approach.

## Experimentation

This study aimed to obtain the most optimal neural architecture by applying a novel search space and search strategies for solving the classification problem defined in  Introduction section. Therefore, the experiment carried out was two-fold: firstly, we experimented with the proposed search space and search strategies to demonstrate the effectiveness of the methods. Secondly, the top-performing CNN architecture obtained from the first experiment was then applied to detect abnormalities in digital histopathology images confirming the presence of breast cancer. This section therefore presents a detailed outline of configurations, parameter values, and characteristics of the histopathological datasets applied in the experimentation.

### Search space configuration

The configuration for generating potential solutions into the search space is presented in this subsection. This configuration is necessary to guide Algorithm 1 to boost the possibility of generating potential solutions (neural networks) for maximizing classification accuracy and minimizing loss in digital histopathology images for the detection of breast cancer. This configuration provides the encoding scheme proposed in this study with a wide range of parameters to generate and encode the possible network topologies to make the search efficient and effective.

The general hyperparameters (GH) block consists of four parameters and is summarized in GH = {G_b_, G_α_, G_e_, G_o_} so that G_b_, G_α_, G_e_, and G_o_ are computed using ${G}_{b}={2}^{n}-1$, ${G}_{\alpha }=rand(1|5)\cdot {10}^{-n}$, ${G}_{o}=O\left[n\right],$ and ${G}_{e}=5$ respectively. Where n = 0, 1, 2 for batch size (G_b_), is represented as random mode = 0, batch mode = 1, mini-batch mode = 3; learning rates (G_α_) is computed by generating random number between 1 and 5 resulting in α = {1 × 10 − 5, 5 × 10 − 5, 1 × 10 − 4, 5 × 10 − 4, 1 × 10 − 3, 5 × 10 − 3, 1 × 10 − 2, 5 × 10 − 2, 1 × 10 − 1, 5 × 10 − 1};n = 1,2,3,4,5 for G_α_; n = 0,1,2,3,4,5,6,7 for G_o_, and O = {0 =  > "SGD", 1 =  > "Adam", 2 =  > "RMSprop", 3 =  > "Adagrad", 4 =  > "Nestrov", 5 =  > "Adadelta", 6 =  > "Adamax", 7 =  > "Momentum", 8 =  > " Nestrov Accelerated Gradient"}. The range of values derivable for the input-Zeropadding block, represented as IZ = { Z_α_}, to determine if input will be zero-padded or not, is shown computed using ${I}_{z}=rand(0|1)$.

Convolutional block (CB) hyperparameters are denoted as follows: CB = {C_*L*_, C_*C*_, C_*AF*_, C_*K*_, C_*F*_, C_*PS*_, C_*PT*_, C_*R*_} and so that C_*L*_, C_*C*_, C_*AF*_, C_*K*_, C_*F*_, C_*PS*_, C_*PT*_, and C_*R*_ are computed using ${C}_{L}=2n-1$, ${C}_{C}=3-n$, ${C}_{AF}=AF\left[n\right], {C}_{K}={2}^{n}$, ${C}_{F}=2+\left(n-1\right)$, ${C}_{PS}=2+n$, ${C}_{PT}=n$, and ${C}_{R}=n$ respectively. Where n = 1,2,3,4 for C_*L*_, to determine the number of blocks of convolutional layers an arbitrary neural network may possess; n = 0, 1, 2 for C_*C*_, to compute convolutional layers in a block; n = 0, 1, 2 for C_*AF*_, and indexes AF = {0 =  > "ReLU"**,** 1 =  > "LeakyReLU", 2** =  > “**Parametric ReLU”}; n = 3, 4, 5, 6, 7, 8, 9, 10 for C_*K*_; n = 0, 2, 4, 6, 8, 10 for C_*F*_; n = 0, 1, 2, for C_*PS*_; n = 0 =  > Max pooling, 1 =  > Average pooling C_*PT*_, n = 0 =  > L1, 1 =  > L2 and 2 =  > L1L2 regularizations for C_*R*_; meanwhile, our configuration allows for the use of padding as *same* and stride = *1* in convolutional layers.

Fully-connected block (FCB) parameters are denoted by FCB = {F_*L*_, F_*AF*_, F_*D*_, F_*R*_} and are computed as follows: F_*L*_, F_*AF*_, F_*D*_, and F_*R*_ using ${F}_{L}=1+n$_,_ ${F}_{AF}=FAF(n)$_,_ ${F}_{D}=\frac{1}{n}$, and ${F}_{R}=n$ respectively, Where n = 0, 1 for computing the number of F_*L*_ flatten operations; n = 0, 1 for obtaining F_*AF*_ which is further defined by indexing: FAF = {0 =  > "softmax"**,** 1 =  > "sigmoid"}; n = 1.0, 1.1…0.2.0, are used in computing F_*D*_; n = 0 =  > L1, 1 =  > L2 and 2 =  > L1L2 regularizations for F_*R*_. The Loss function block denoted by LF has only one element: {LF_*L*_} where loss function for the search space can be drawn from the {categorical cross-entropy, sparse-cross-entropy} when n = 0 and 1, respectively.

The summary presented in Table 4 identifies the collection of possible values derivable for the search space in configuring potential CNN architectures. The EOSA algorithm is also configured and experimented with the parameters listed in Table 5. The following sub-section presents the configuration of the environment for the experiment.

**Table 4:** A summary of formula for computing values for hyperparameters and the corresponding search space using the proposed encoding scheme.

Hyperparameter	Formula	Hyperparameter search space
G_b_	${2}^{n}-1$	[0, 1, 3]
G_α_	$rand(1|5)\cdot {10}^{-n}$	[1 × 10 − 5, 5 × 10 − 5, 1 × 10 − 4, 5 × 10 − 4, 1 × 10 − 3, 5 × 10 − 3, 1 × 10 − 2, 5 × 10 − 2, 1 × 10 − 1, 5 × 10 − 1]
G_o_	$O[n]$	[0 = > "SGD", 1 = > "Adam", 2 = > "RMSprop", 3 = > "Adagrad", 4 = > "Nestrov", 5 = > "Adadelta", 6 = > "Adamax", 7 = > "Momentum]
G_e_	$5$	5
I_Z_	$rand(0|1)$	[0,1]
C_*L*_	$2n-1$	[1, 3, 5, 7, 9, 11]
C_*C*_	$3-n$	[1, 2, 3]
C_*AF*_	$AF[n]$	[0 = > "ReLU"**,** 1 = > "LeakyReLU", 2** = > “**Parametric ReLU”]
C_*K*_	${2}^{n}$	[8, 16, 32, 64, 128, 256, 512, 1024]
C_*F*_	$2+\left(n-1\right)$	[1, 3, 5, 7, 9, 11]
C_*PS*_	$2+n$	[2, 3, 4]
C_*PT*_	$n$	[Max pooling, Average pooling]
C_*R*_	$n$	[ L1, L2, L1L2]
F_*L*_	$1+n$	[1, 2]
F_*AF*_	$FAF(n)$	[0 = > " Softmax"**,** 1 = > " Sigmoid "]
F_*D*_	$\frac{1}{n}$	[0.35, 0.4, 0.45, 0.5]
F_*R*_	$n$	[L1, L2, L1L2]
LF_*L*_	$n$	[categorical cross-entropy, sparse cross-entropy]

**Table 5:** Notations and description for variables and parameters used for experimenting with EOSA optimization algorithm.

Symbols	Descriptions	Range
Epoch	Number of iteration for the EOSA algorithm	5
Population	Number of neural architectures in the search space	50
π	Recruitment rate of susceptible human individuals	Variable
ŋ	Decay rate of Ebola virus in the environment	(0, ∞)
α	Rate of hospitalization of infected individuals	(0, 1)
Γ	Disease-induced death rate of human individuals	[0.4, 0.9]
β_1_	Contact rate of infectious human individuals	Variable
β_2_	Contact rate of pathogen individuals/environment	Variable
β_3_	Contact rate of deceased human individuals	Variable
β_4_	Contact rate of recovered human individuals	Variable
γ	Recovery rate of human individuals	(0, 1)
τ	Natural death rate of human individuals	(0, 1)
δ	Rate of burial of deceased human individuals	(0, 1)
ϑ	Rate of vaccination of individuals	(0, 1)
ϖ	Rate of response to hospital treatment	(0, 1)
μ	Rate response to vaccination	(0, 1)
ξ	Rate of quarantine of infected individuals	(0, 1)

### Configuration for experimentation environment

Exhaustive experimentation done for evaluating the proposed EOSA, described in Algorithm 1, was carried out in a workstation environment with the following configurations: Intel (R) Core i5-7500 CPU 3.40 GHz, 3.41 GHz; RAM of 16 GB; and 64-bit Windows 10 OS for each configuration of the system on the network. Similarly, those for the neural architecture search and for convolutional and classification processes were carried out in the same computational environment.

### Experimentation dataset

This study is focused on applying the experimentation of the proposed NAS model on digital histopathological images. We allowed every candidate CNN architecture to be evaluated using these images for performance evaluation. We chose the publicly available benchmark datasets, namely BACH^57^ and BreakHis^58,59^. The motive for choosing these datasets was to provide sufficient data for the experimentation and allow for the reproducibility of the proposed approach. The experiments were staged in two (2): generating and searching for best performing networks, and the second experiment for full training of top-5 networks. As a result, we rigorously applied the datasets to the top-performing CNN architecture resulting from the stage 1 experiment.

**Figure 3:**
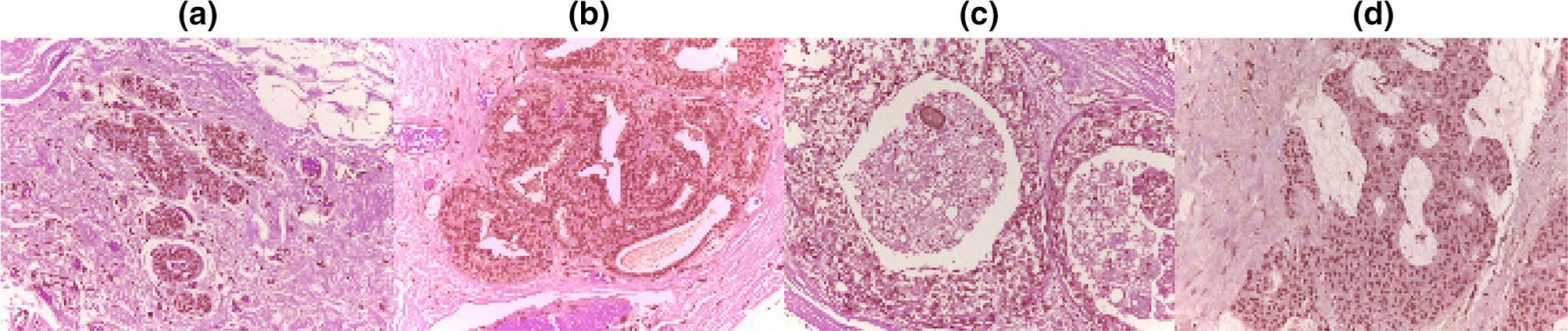
Sample images from the BACH datasets showing (**a**) normal (**b**) benign (**c**) in situ carcinoma and (**d**) invasive carcinoma cases.

**Figure 4:**
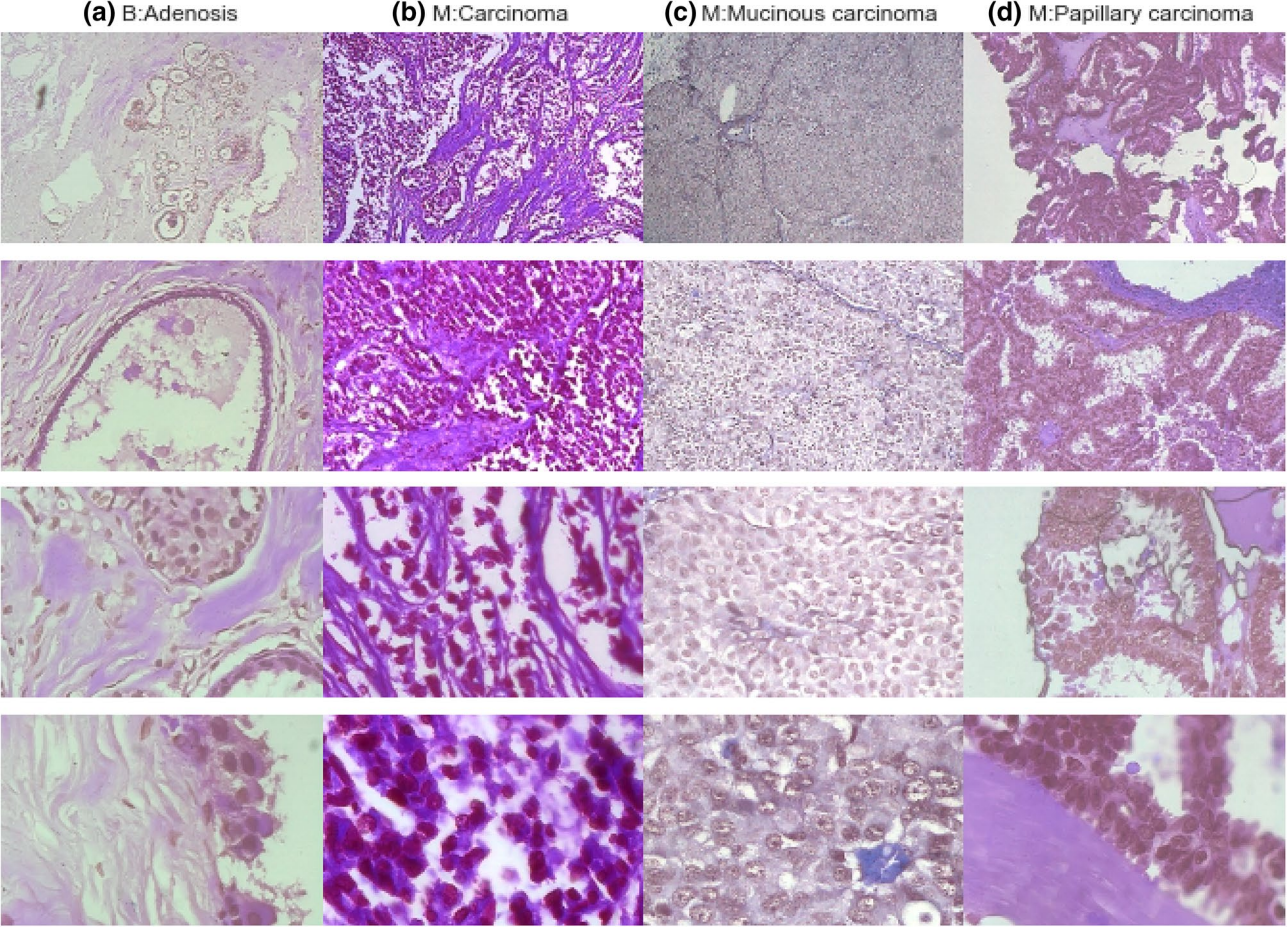
Sample images from the BreakHis datasets showing (**a**) adenosis, (**b**) ductal carcinoma, (**c**) mucinous carcinoma, and (**d**) papillary carcinoma malignant cases. Each column shows the magnification of samples for (**a**)–(**d**) in 40X, 100X, 200X, and 400X accordingly. The H&E stain the nuclei with a dark purple (Hematoxylin) and the cytoplasm with a light pink (Eosin).

The image samples obtained from the BACH and BreakHis datasets were further resized to sizes 224 × 224 to allow for input into the neural architectures and the top-performing neural network architectures. This resizing became necessary because the original image size from BACH was 2048 × 1536 pixels and consisted of 400 Hematoxylin and eosin (HE) stained images, while the BreakHis dataset contained a total of 9,109 (actually 7,909 samples after removal of tissue samples) microscopic images with an image size of 700 × 460 pixels. The classes of images obtained from BACH are normal, benign**, **in situ carcinoma or invasive carcinoma, while those of BreakHis are categorized as benign or malignant. The benign and malignant samples of BreakHis are further categorized into adenosis (A), fibroadenoma (F), phyllodes tumor (PT), and tubular adenona (TA) as benign; and carcinoma (DC), lobular carcinoma (LC), mucinous carcinoma (MC) and papillary carcinoma (PC) as malignant. Figures 3 and 4 show some samples drawn from BACH and BreakHis datasets respectively.

According to their classes, the breakdown for the BACH image samples are: 100 samples of normal, 100 samples of benign, 100 samples of in situ carcinoma, and 100 samples of invasive carcinoma. Similarly, the BreakHis datasets image samples contain 2,480 benign and 5,429 malignant samples. Both the BACH and BreakHis datasets image samples are 3-channelled RGB. Also, we discovered that the magnification for the BACH dataset is 200 × , and those of BreakHis were presented at 40X, 100X, 200X, and 400Xmagnifications. We, however, preprocessed the images to allow for resizing and elimination of potential errors arising from the stain on the raw inputs. We applied the basic operations of reduction of background noise and image enhancement. Furthermore, we applied image normalization operations from Reinhard^60^ and Macenku^61^ to normalize our histopathology images.

## Results and discussion

In this section, the result of the experimentation is presented, and the findings are discussed. Two categories of results are considered: performance of the EOSA algorithm as compared with four similar metaheuristic algorithms and the performance of the NAS model in obtaining best performing CNN architecture.

### Performance evaluation of EOSA metaheuristic algorithm

The EOSA experiment was carried out using 25 benchmark optimization functions listed in Table 6. These same functions were applied to artificial bee colony (ABC), whale optimization algorithm (WOA), particle swarm optimization (PSO), and genetic algorithm (GA) metaheuristic algorithms. Each of these optimization algorithms was executed for 500 epochs and 20 runs for stability. The result of the experimentation is listed in Table 7.

**Table 6:** Standard and CEC benchmark functions used for the experimentation in evaluating the performances of EOSA, ABC, WOA, PSO and GA.

ID	Function name	Model of the function
F1	Ackley	$f\left(x\right)=-20{e}^{\left(-0.2 \sqrt{\frac{1}{n}{\sum }_{1}^{n}{x}_{1}^{2}}\right) }- -{e}^{\left(\frac{1}{n}{\sum }_{1}^{n}\mathrm{cos}(2\pi {x}_{i}) \right)}+20+{e}^{(1)}$
F2	Alpine	$f\left(x\right)=\sum_{i=1}^{n}\left|{x}_{i}\mathrm{sin}\left({x}_{i}\right)+0.1{x}_{i}\right|$
F3	Brown	$f\left(x\right)=\sum_{i=1}^{n-1}{{({x}_{i}^{2})}^{\left({x}_{i+1}^{2}+1\right)}+({x}_{i+1}^{2})}^{({x}_{i}^{2}+1)}$
F4	Bent Cigar	${f}_{20}\left(x\right)={x}_{1}^{2}+{10}^{6}\sum_{i=2}^{D}{x}_{i}^{2}$
F5	Dixon and Price	${f}_{18}\left(x\right)={10}^{6}{x}_{1}^{2}\sum_{i=2}^{D}{x}_{i}^{2}$
F6	Discus Function	$f\left(x\right)={({x}_{1}-1)}^{2}+ \sum_{i=2}^{n}i{(2{x}_{i}^{2}- {x}_{i-1})}^{2}$
F7	Levy	${f}_{12}\left(x\right)=\sum_{i=1}^{n}{({x}_{i}-1)}^{2}\left[{sin}^{2}(3\pi {x}_{i+1})\right]+{sin}^{2}\left(3\pi {x}_{1}\right)+ |{x}_{n}-1|\left[1+ {sin}^{2}(3\pi {x}_{n})\right]$
F8	Powel	$f\left(x\right)={\left({x}_{1}+10{x}_{2}\right)}^{2}+5{({x}_{3}+{x}_{4})}^{2}+{({x}_{2}- {2x}_{3})}^{4}+10{({x}_{1}-{x}_{4})}^{4}$
F9	Quartic	${f}_{6}\left(x\right)=\sum_{i=1}^{n}{ix}_{i}^{4}$
F10	Rastrigin	${f}_{9}\left(x\right)=\sum_{i=1}^{n}[{x}_{i}^{2}-10\mathrm{cos}\left(2\pi {x}_{i}\right)+10]$
F11	SR-F27	Shifted and Rotated Rastrigin’s Function
F12	Wavy 1	$f\left(x\right)=\sum_{i=1}^{n}{x}_{i}^{2}+{({\sum }_{i=1}^{n}0.5i{x}_{i})}^{2}+{({\sum }_{i=1}^{n}0.5i{x}_{i})}^{4}$
F13	Zakharov	$f(x)=\frac{1}{n}\sum_{i=1}^{n}1-\mathrm{cos}(10{x}_{i}){e}^{-\frac{1}{2}{x}_{i}^{2}}$
F14	Salomon	${f}_{19}\left(x\right)=1-cos\left(2\pi \sqrt{\sum_{i=1}^{n}{x}_{i}^{2}}\right)+0.1\sqrt{\sum_{i=1}^{n}{x}_{i}^{2}}$
F15	Weierstrass Function	$f\left(x\right)=\sum_{i=1}^{D}{({\sum }_{i=0}^{20}[{0.5}^{k}\mathrm{cos}(2\pi . {3}^{k}({x}_{i}+0.5))])}$

**Table 7:** Comparison of best, worst, mean, median and standard deviation (stdev) values for EOSA, ABC, WOA, PSO, and GA metaheuristic algorithms using the classical benchmark and IEEE CEC functions over 500 epochs and 100 population size.

Functions	Metrics	EOSA	ABC	WOA	PSO	GA
F1	Best	**0.046173**	0.046591	0.046596	0.046571	9.94223
	Worst	0.046588	20.8892	0.046596	**0.046571**	19.83618
	Mean	0.046465	19.30266	0.046596	0.046571	10.40362
	Median	0.046512	19.15063	0.046596	0.046571	10.1534
	Stdev	0.000107	0.948262	5.20E−18	5.55E−18	0.938523
F2	Best	**0.002556**	0.0028	0.002748	0.002769	39.73652
	Worst	0.002768	245.4735	**0.002748**	0.002769	184.0994
	Mean	0.002608	33.16789	0.002748	0.002769	44.36342
	Median	0.002607	7.26278	0.002748	0.002769	42.07979
	Stdev	4.68E−05	52.19852	3.69E−19	2.82E−19	10.53887
F3	Best	**8.68E**−**05**	0.000417	0.000416	0.000414	921.248
	Worst	**0.000405**	1498.884	0.000416	0.000414	1269.038
	Mean	0.00011	294.4233	0.000416	0.000414	938.3754
	Median	8.86E−05	203.1162	0.000416	0.000414	929.879
	Stdev	4.55E−05	227.7159	6.23E−20	7.86E−20	30.31403
F4	Best	**1.39E**−**12**	2.49E−12	2.45E−12	2.49E−12	4.13E + 09
	Worst	2.48E−12	2.57E + 11	**2.45E**−**12**	2.49E−12	1.34E + 11
	Mean	2.05E−12	2.05E + 11	2.45E−12	2.49E−12	5.68E + 09
	Median	2.18E−12	2.01E + 11	2.45E−12	2.49E−12	4.45E + 09
	Stdev	3.79E−13	1.3E + 10	3.03E−28	4.04E−28	7.3E + 09
F5	Best	**9.30E**−**13**	2.78E−12	2.80E−12	2.79E−12	395.2324
	Worst	2.86E−12	43,618,954	2.80E−12	**2.79E**−**12**	194,298
	Mean	1.17E−12	161,597.3	2.80E−12	2.79E−12	2351.452
	Median	9.35E−13	1152.776	2.80E−12	2.79E−12	423.69
	Stdev	4.16E−13	2,214,592	4.04E−28	3.03E−28	12,218
F6	Best	**4.07E**−**11**	1.02E−10	1.02E−10	1.02E−10	6952.905
	Worst	**1.02E**−**10**	1,342,862	**1.02E**−**10**	**1.02E**−**10**	195,495.6
	Mean	7.19E−11	263,974.3	1.02E−10	1.02E−10	14,746.75
	Median	7.20E−11	253,737.4	1.02E-10	1.02E−10	8375.828
	Stdev	2.03E−11	63,079.51	1.62E−26	1.81E−26	21,265.92
F7	Best	**5.25E**−**05**	0.000248	0.000248	0.000251	41.79268
	Worst	0.000253	1479.208	**0.000248**	0.000251	823.37
	Mean	0.0002	106.1467	0.000248	0.000251	58.77442
	Median	0.000228	15.67991	0.000248	0.000251	47.54116
	Stdev	6.14E−05	232.7978	4.20E−20	4.74E−20	50.30075
F8	Best	**8.19E**−**06**	1.98E−05	2.41E−05	2.31E−05	0.009794
	Worst	**2.11E**−**05**	24.42778	2.41E−05	2.31E−05	5.436187
	Mean	1.32E−05	0.345815	2.41E−05	2.31E−05	0.038439
	Median	1.14E−05	0.005065	2.41E−05	2.31E−05	0.013349
	Stdev	4.71E−06	1.7694	3.22E−21	4.40E−21	0.279212
F9	Best	**5.08E**−**11**	1.38E−10	1.40E−10	1.39E−10	30,500.52
	Worst	1.40E−10	3.68E + 09	1.40E−10	**1.39E**−**10**	1.13E + 09
	Mean	9.97E−11	2.53E + 09	1.40E−10	1.39E−10	4,511,122
	Median	1.06E−10	2.44E + 09	1.40E−10	1.39E−10	144,930.2
	Stdev	3.33E−11	2.26E + 08	1.29E−26	1.94E−26	54,440,104
F10	Best	**0.000153**	0.000471	0.000474	0.000475	745.3493
	Worst	0.000475	1599.605	**0.000474**	0.000475	1278.155
	Mean	0.000287	444.8808	0.000474	0.000475	772.7753
	Median	0.00028	315.5723	0.000474	0.000475	760.9054
	Stdev	0.000134	271.854	7.32E−20	8.40E−20	45.18663
F11	Best	**0.000322**	0.000331	0.000333	0.00033	1654.473
	Worst	**0.000331**	2490.439	0.000333	0.00033	2194.09
	Mean	0.000326	1912.671	0.000333	0.00033	1676.178
	Median	0.000325	1851.11	0.000333	0.00033	1664.138
	Stdev	3.15E−06	159.2676	4.88E−20	3.79E−20	45.46335
F12	Best	**1.98E**−**30**	2.00E−29	1.82E−29	2.01E−29	112,016.4
	Worst	1.96E−29	2.76E + 24	**1.82E**−**29**	2.01E−29	1.92E + 24
	Mean	5.07E−30	1.12E + 22	1.82E−29	2.01E−29	8.29E + 21
	Median	2.13E−30	8.34E + 17	1.82E−29	2.01E−29	140,116
	Stdev	4.89E−30	1.42E + 23	2.70E−45	3.22E−45	1.06E + 23
F13	Best	0.303455	0.30833	**0.245368**	0.307142	2.686451
	Worst	**0.306781**	2.842985	0.306802	0.307142	2.778775
	Mean	0.304267	1.791644	0.261832	0.307142	2.686881
	Median	0.304119	1.67436	0.245368	0.307142	2.686451
	Stdev	0.00089	0.256993	0.023673	3.61E−17	0.005805
F14	Best	**5.40E**−**06**	2.46E−05	2.45E−05	2.44E−05	412.1038
	Worst	**2.44E**−**05**	25,843.77	2.45E−05	**2.44E**−**05**	13,787.81
	Mean	1.74E−05	21,080.93	2.45E−05	2.44E−05	580.0391
	Median	1.99E−05	20,736.46	2.45E−05	2.44E−05	459.1532
	Stdev	6.69E−06	1251.021	3.22E−21	2.20E−21	760.3748
F15	Best	**0.005718**	0.005899	0.005866	0.005885	14.62603
	Worst	0.005876	130.4765	**0.005866**	0.005885	97.42765
	Mean	0.005761	30.95515	0.005866	0.005885	16.72031
	Median	0.005757	7.717655	0.005866	0.005885	15.10426
	Stdev	4.67E−05	39.61793	6.07E−19	8.67E−19	6.393344

Table 7 shows that EOSA had the lowest values for the best solutions using the F1–F12 and F14–F15 compared with the best solutions for ABC, WOA, PSO and GA. Although PSO maintained a lead only in F13 compared to EOSA, ABC, WOA, and GA, the performance margin was small compared with EOSA, and EOSA showed superiority in fourteen (14) of fifteen (15) functions evaluated. Also, EOSA yielded a significant performance compared with ABC, WOA, PSO and GA based on the values of worst solutions for F1–F15. Table 8 shows that the EOSA performed well in the solutions obtained for the constrained IEEE CEC-2017 benchmark functions compared to other competing algorithms. The EOSA had obtained a total of eight (8) best results out of the nine (9) functions.

**Table 8:** Comparison of best, worst, mean, median and standard deviation (stdev) values for EOSA, ABC, WOA, PSO, and GA metaheuristic algorithms using the constrained IEEE CEC-2017 benchmark functions over 500 epochs and 100 population size.

Functions	Metrics	EOSA	ABC	WOA	PSO	GA
**CEC01**	Best	**2.75E**−**11**	2.78E−11	2.78E−11	2.78E−11	6,500,451
	Stdev	**1.46E**−**15**	8.44E + 08	2.78E−11	3.88E−27	3.10E + 08
	Median	2.75E−11	4.97E + 09	3.39E−27	2.78E−11	17,405,089
**CEC02**	Best	**2.48E**−**12**	2.49E−12	2.45E−12	2.49E−12	4.17E + 09
	Stdev	9.11E−17	1.30E + 10	2.45E−12	2.83E−28	7.53E + 09
	Median	2.48E−12	2.02E + 11	4.64E−28	2.49E−12	4.44E + 09
**CEC03**	Best	**1.01E**−**10**	1.02E−10	1.02E−10	1.03E−10	8666.065
	Stdev	3.19E−14	124,317.8	1.02E−10	2.13E−26	23,773.8
	Median	1.01E−10	251,561	1.36E−26	1.03E−10	12,804.61
**CEC04**	Best	3.71E−12	**3.68E**−**12**	3.70E−12	3.73E−12	1,099,091
	Stdev	1.35E−16	9.64E + 09	3.70E−12	7.88E−28	2.26E + 09
	Median	3.71E−12	8.50E + 10	5.65E−28	3.73E−12	5,359,283
**CEC05**	Best	**0.045669**	0.045719	0.045711	0.045704	18.25292
	Stdev	9.25E−07	0.957905	0.045711	5.90E−18	0.531952
	Median	0.045669	20.02451	5.90E−18	0.045704	18.40464
**Shift CEC06**	Best	0.001299	0.001302	**0.001298**	0.001298	618.1048
	Stdev	8.59E−09	31.98589	0.001298	1.63E−19	6.159877
	Median	0.001299	710.5576	1.52E−19	0.001298	619.0061
**Shift CEC07**	Best	**0.000224**	0.000228	0.000227	0.000226	761.7748
	Stdev	7.57E−09	141.8918	0.000227	3.25E−20	66.00672
	Median	0.000224	2526.215	4.07E−20	0.000226	766.6583
**Shift CEC08**	Best	**0.000343**	0.000345	0.000344	0.000343	1557.367
	Stdev	4.04E−08	156.4804	0.000344	3.52E−20	44.58426
	Median	0.000343	1756.355	3.79E−20	0.000343	1567.959
**Shift-rotate CEC08**	Best	**0.000333**	0.00033	0.000334	0.000332	1657.835
	Stdev	1.49E−08	158.7789	0.000334	4.88E−20	45.19802
	Median	0.000333	1857.348	4.34E−20	0.000332	1671.562
**Shift CEC09**	Best	**2.16E**−**05**	2.17E−05	2.19E−05	2.18E−05	22,425.71
	Stdev	2.54E−09	3268.026	2.19E−05	1.86E−21	1633.723
	Median	2.16E−05	21,565.89	2.20E−21	2.18E−05	22,826.18

**Figure 5:**
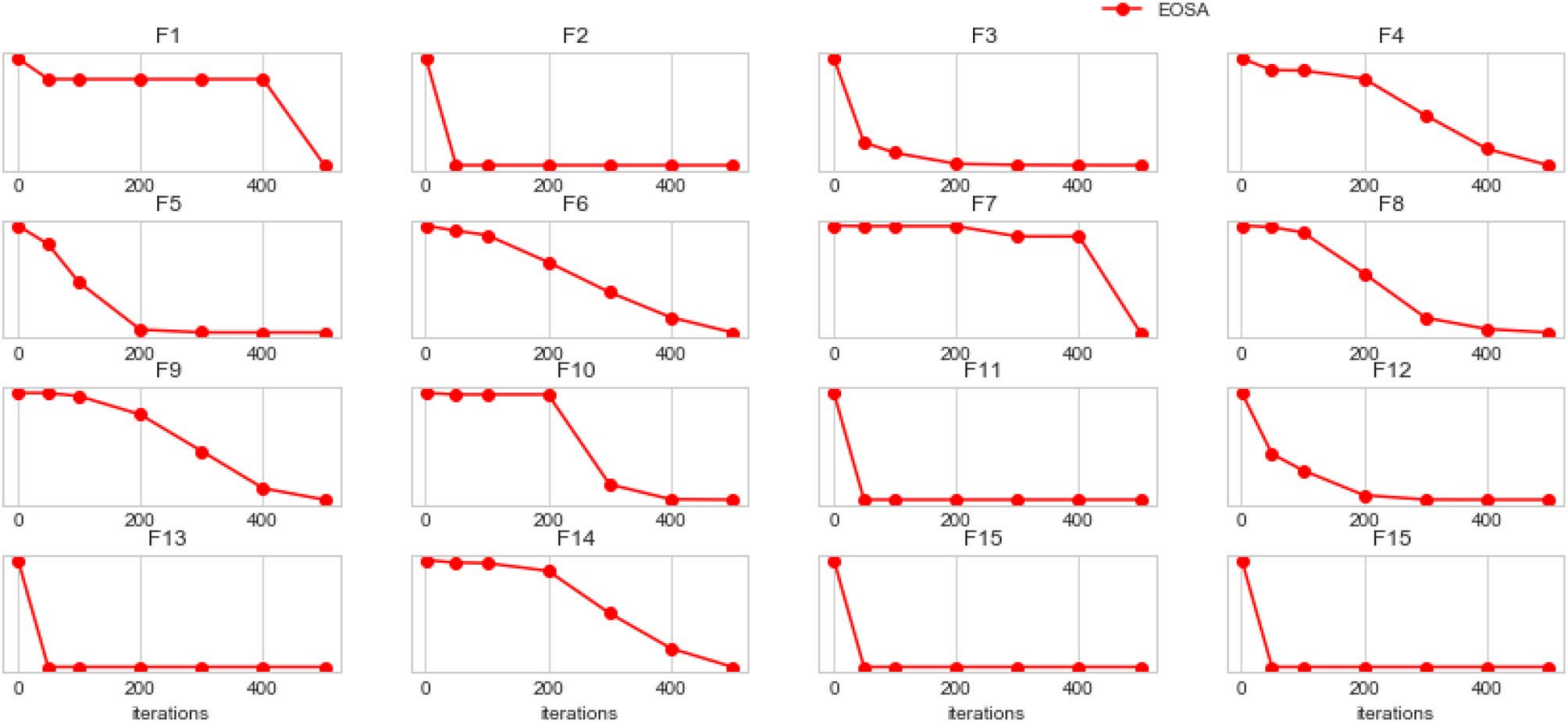
Convergent curves of EOSA optimization algorithm on F1, F2, F3, F4, F5, F6, F7, F8, F9, F10, F11, F12, F13, F14 and F15 standard benchmark functions.

**Figure 6:**
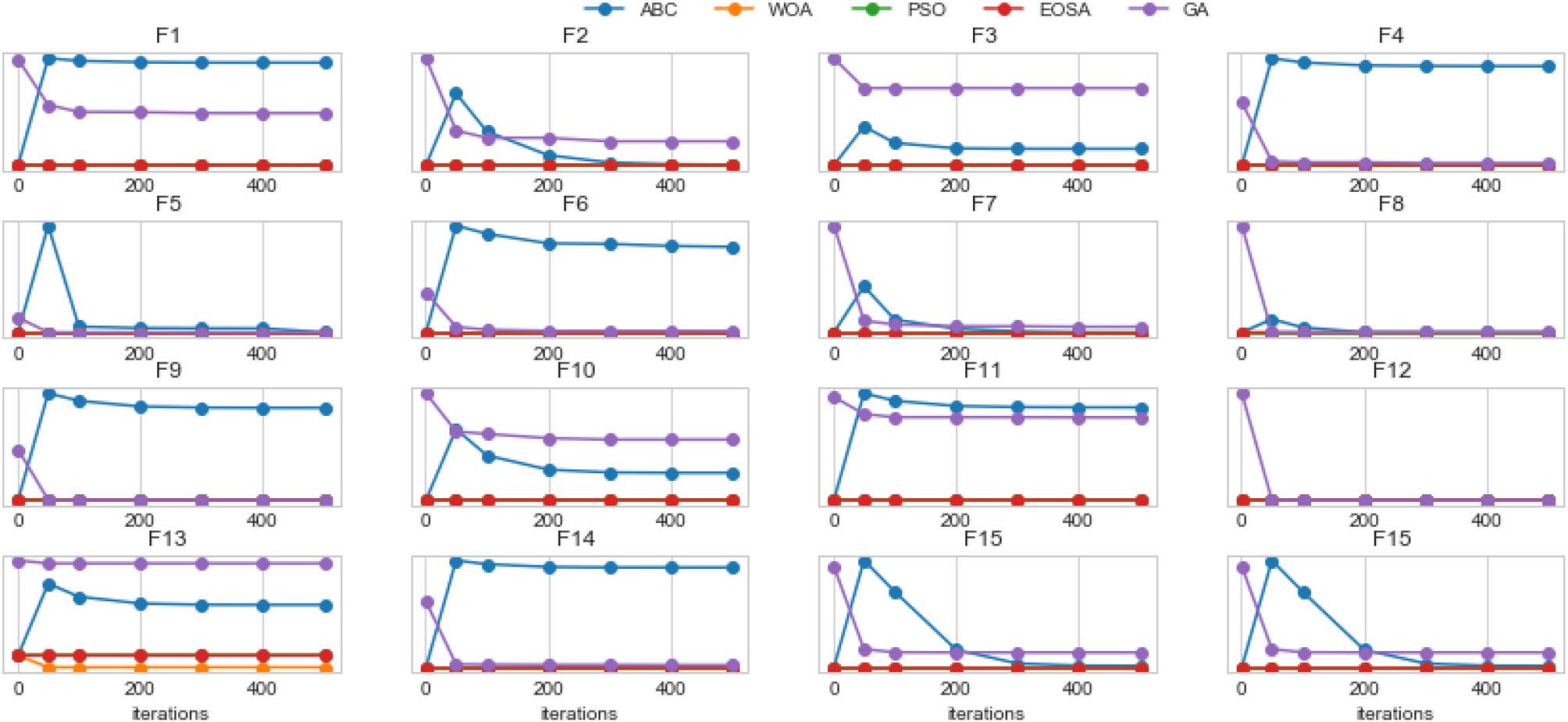
Comparison of convergence curves of the performance of EOSA, ABC, WOA, PSO, and GA optimization algorithms on all standard benchmark functions applied in this study.

**Figure 7:**
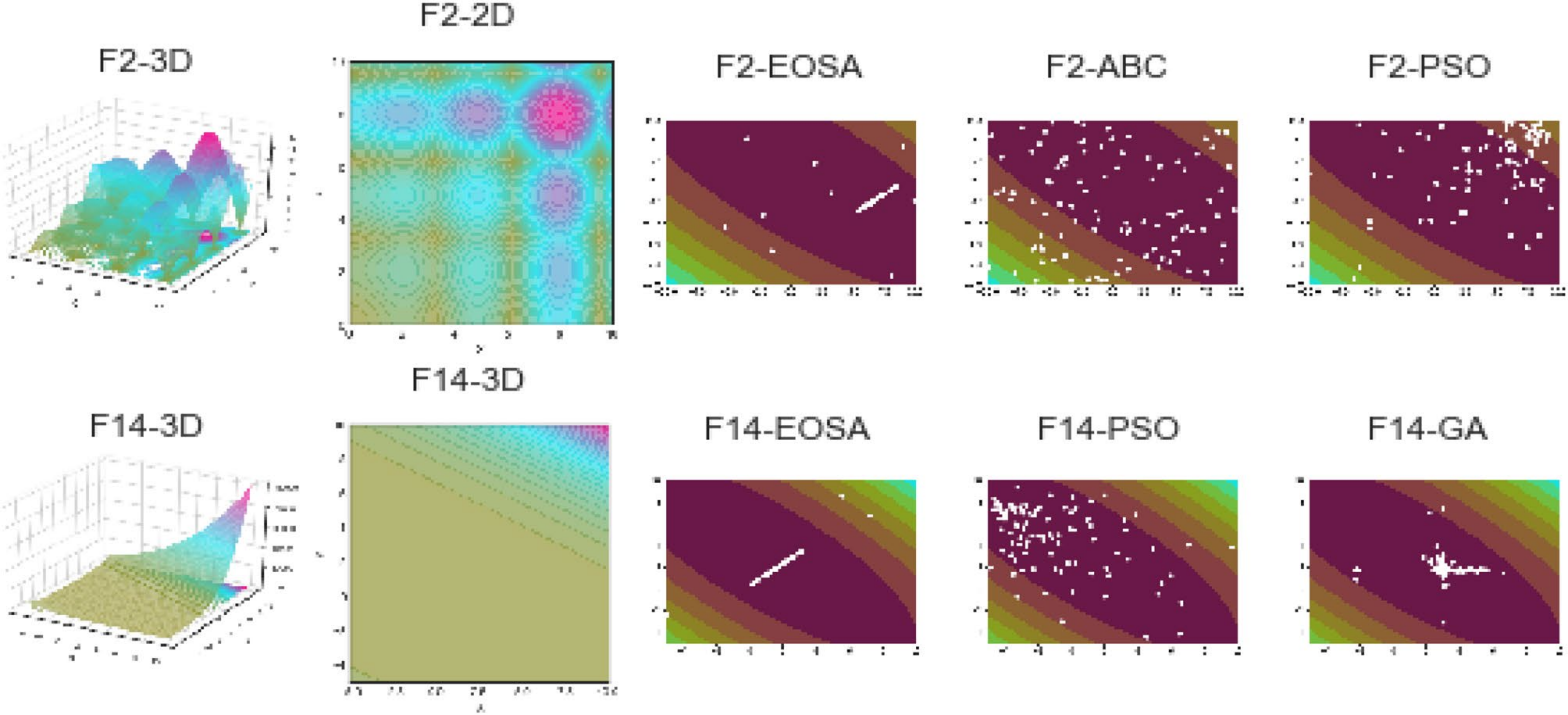
Comparison of convergence curves of the performance of EOSA, ABC, WOA, PSO, and GA optimization algorithms on all standard benchmark functions applied in this study.

Figures 5 and 6 illustrate the convergence of EOSA on F1–F15 and convergence of EOSA compared with ABC, WOA, PSO and GA on F1–F15, respectively. The plots in Fig. 5 confirm that the convergence of EOSA is impressive though the significance of its convergence has been overshadowed in Fig. 6 due to variation of values. Also, we observed the convergence of each solution for EOSA, ABC, WOA, PSO, and GA using a scatter plot. The outcome, as shown in Fig. 7, aligns with the graphs in Figs. 5 and 6. The results show that the EOSA algorithm is a candidate optimization algorithm capable of sufficiently learning the problem of automating the design of CNN architectures for the search strategy of a NAS model. Furthermore, the results guarantee that EOSA can compete with state-of-the-art optimization algorithms.

Now that the performance of EOSA as a metaheuristic algorithm was confirmed to be suitable for optimizing the search strategy of a NAS model, we proceeded to experiment using it in the NAS model experimentation. The result of this experiment is presented and discussed in the next section.

### Performance evaluation of CNN design using EOSA-NAS

**Figure 8:**
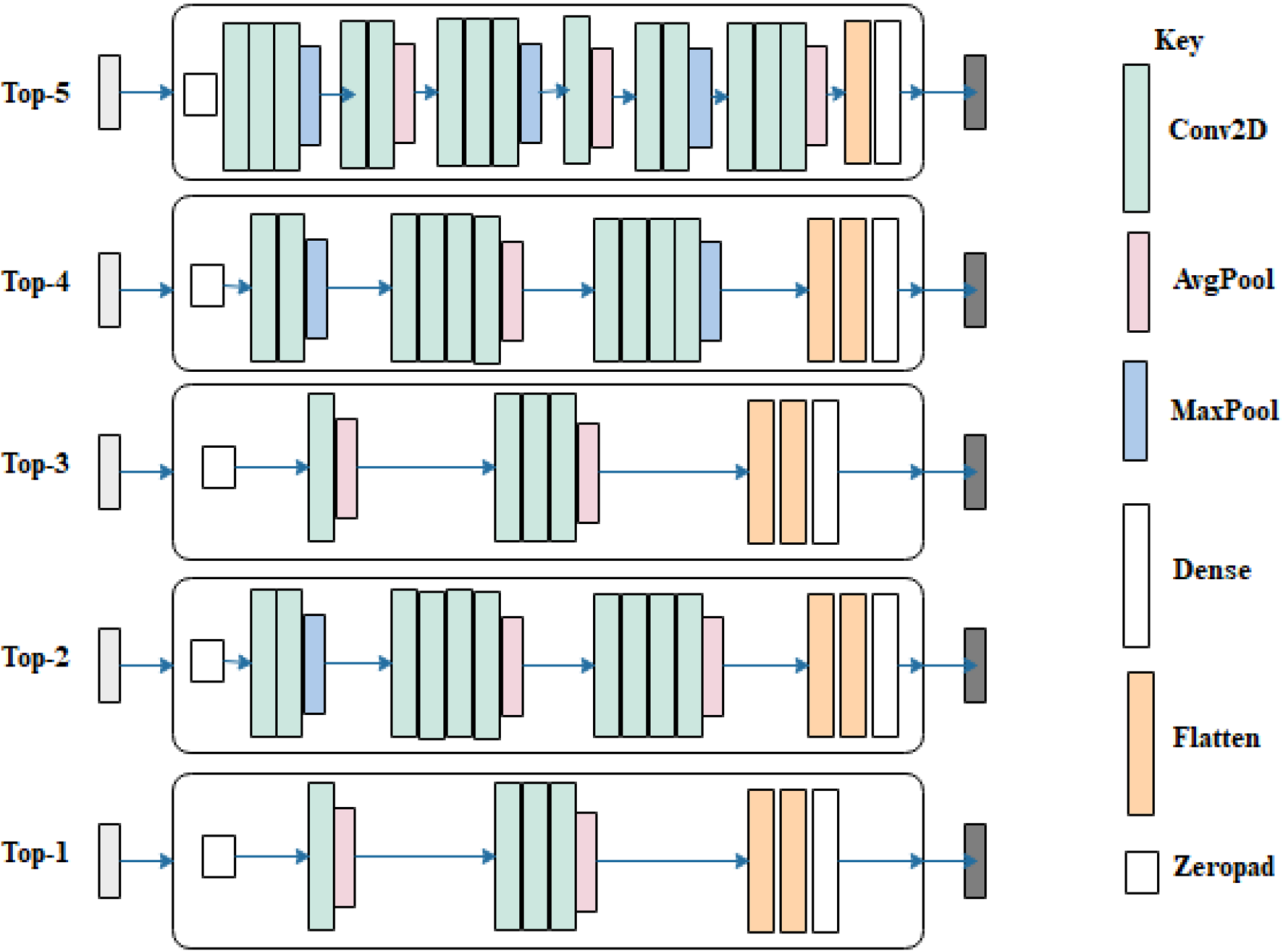
Neural network architectures of the Top-5 generated network architectures generated for the search space.

The initial solutions (CNN architectures) generated into the search space were optimized using the EOSA algorithm during the search strategy stage of the NAS model. The optimization in EOSA was executed for 500 epochs, and the configuration of each solution was reevaluated using the evaluation strategy of our NAS model. The optimized CNN architectures were logged for each iteration, while the final configurations for all the CNN architectures were examined and used for the result presented in this section. Table 9 presents the configurations of the top-5 CNN architectures, and their network topologies are shown in Fig. 8.

**Table 9:** Comparison of parameters for the best five (5) initial neural network configurations (solutions) generated for the search space.

Parameters	Top-1	Top-2	Top-3	Top-4	Top-5
Dataset batching	Random sample size	Half of dataset	Random sample size	Half of dataset	Random sample size
Zero padding	Yes	Yes	Yes	Yes	Yes
No. Convo-Pool blocks	2	3	2	3	6
Details of Convolution layers	[1Convo, 'relu', 32, 9, 2, 'Avg', 'L1'], [3Convo, 'relu', 64, 9, 2, 'Avg', 'L1']	([3Convo, 0.005, 'Adagrad', 3], True, [2, 'relu', 32, 3, 2, 'Max', 'L1'], [4, 'relu', 64, 3, 2, 'Avg', 'L1'], [4, 'relu', 128, 3, 2, 'Avg', 'None'],	[1Convo, 'relu', 32, 9, 2, 'Avg', 'L1'], [3Convo, 'relu', 64, 9, 2, 'Avg', 'L1']	[2Convo, 'relu', 32, 3, 2, 'Max', 'None'], [4, 'relu', 64, 3, 2, 'Avg', 'None'], [4, 'relu', 128, 3, 2, 'Max', 'L1']	[3Convo, 'relu', 32, 9, 2, 'Max', 'L1'], [2, 'relu', 64, 1, 2, 'Avg', 'None'], [3, 'relu', 128, 11, 2, 'Max', 'None'], [1, 'relu', 256, 9, 2, 'Avg', 'L1'], [2, 'relu', 512, 7, 2, 'Max', 'None'], [3, 'relu', 1024, 3, 2, 'Avg', 'None']
Pool size	2 × 2	2 × 2	2 × 2	2 × 2	2 × 2
Filters size	9 × 9, 9 × 9	3 × 3, 3 × 3, 3 × 3	9 × 9, 9 × 9	3 × 3, 3 × 3, 3 × 3	9 × 9, 1 × 1, 11 × 11, 9 × 9, 7 × 7, 3 × 3
Filter count	32 × 32, 64 × 64	32 × 32, 64 × 64, 128 × 128	32 × 32, 64 × 64	32 × 32, 64 × 64, 128 × 128	32 × 32, 64 × 64
No. FC layers	2	3	2	3	1
Dense Layer activation function and dropout rate	Softmax and 0.48	Softmax and 0.5 and LI	Softmax and 0.5	Softmax and 0.45 and L1	Softmax and 0.47 and L1
Learning rate	0.05	0.005	0.05	0.005	1e-05
Optimizer	RMSprop	Adagrad	RMSprop	Adagrad	Adam
Classifier	Categorical crossentropy	Categorical crossentropy	Categorical crossentropy	Categorical crossentropy	Categorical crossentropy

In Table 9, a detailed definition of each of the top five (5) architectures is outlined. Similarly, a graphical illustration of the architectures is shown in Fig. 8. We found that the Top-1 architecture represents a minimal utilization of convolutional and pooling operations while the Top-5 architecture has more of these operations. For instance, the Top-1 has two convolutional blocks with a single convo operation in a block and three convo operations in the second block. In contrast, the Top-5 has 6 convolutional blocks with mostly three convo operations combined with either max or average pooling operations. Another interesting outcome of the resulting top five architectures is that we found a structural similarity between the Top-1 compared with Top-3 and another variation of structural similarity between the Top-2 and Top-4 architectures.

However, in Table 9, we observed that whereas these similarities exist in the structural view of the architectures, there are some significant variations in their detailed implementations. For instance, we found that the three convolutional blocks of the Top-2 architecture have the Max-Avg-Avg pooling operations and the Top-4 Max-Avg-Max pooling operations. In addition, the second convolutional block of the Top-2 architecture allows the use of the L1 (weight decay) network regularizer, whereas that of Top-4 uses none. The reverse of this arrangement is seen in the third convolutional block.

The result in Table 10 shows that the Top-1 architecture achieved a good performance during the 250 iterations as its accuracy for best, mean and median are 0.655, 0.415, and 0.417, respectively. This is a distance from the Top-5, which maintained the values of 0.551, 0.313, and 0.332 for best, mean and median, respectively. We found a similar trend as shown in the results of Top-2, Top-3, and Top-4 performing architectures. The interpretation of these variations informs us that the Top-1 architecture learned the classification problem very well compared to the remaining four (4) architectures. Using a radar chart, we plotted the performance of the top five (5) network architectures using the resulting values of their best, mean, median, worst, and standard deviation. Radar charts provide a good way for visualizing comparisons of data of related attributes or variables which are displayed along their axis.

**Table 10:** Performance comparison for training the five (5) best performing CNN architectures from EOSA-NAS algorithm using mean, median, accuracy and standard deviation for accuracy, and loss, computation time values for the 250 epochs of EOSA.

S/N	Accuracy					Loss			
	Best	Mean	Median	Worst	Stdev	Worst	Median	Best	Latency
Top-5	0.551	0.313	0.332	0.030	0.247	2.79E + 09	1.84	1.84	12.87
Top-4	0.573	0.376	0.359	0.111	0.097	9.13E + 08	3.16	1.31	12.52
Top-3	0.613	0.354	0.326	0.136	0.137	5.1E + 09	2.21	1.318	21.26
Top-2	0.627	0.396	0.350	0.098	0.051	26,261,178	2.21	1.231	39.21
Top-1	0.655	0.415	0.417	0.147	0.150	23,565.56	11,137.88	1.297	93.59

**Figure 9:**
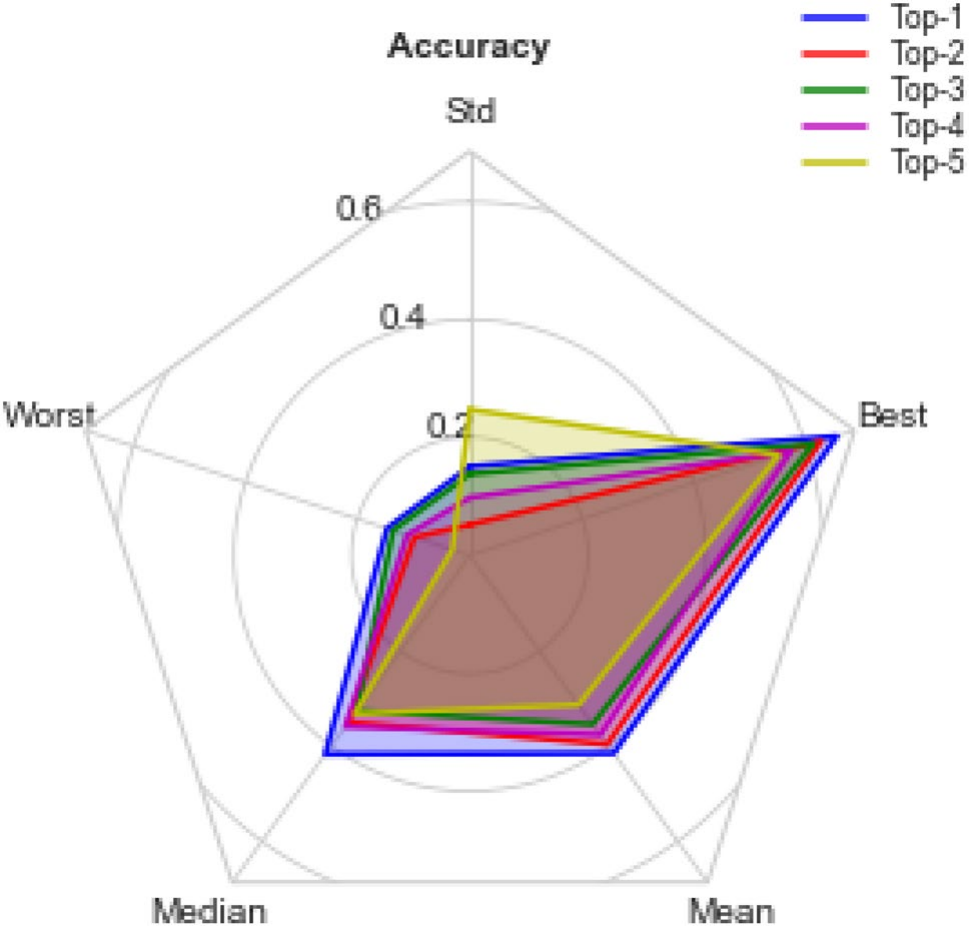
A radar plot showing the performance comparison of the top-5 best performing network architectures from EOSA-NAS algorithm based on mean, median, worst, and best accuracy values.

In Fig. 9, we see that the overall difference in visual representation is apparent by the size and shape of the polygons’ pointing. The polygons point to the best axis more closely because the top5 architectures have their highest accuracy within this variable. The nearness of the polygons’ closeness to the axis is followed by those of *mean* and *median* variables, confirming the distribution of accuracies for the top-5 architectures within those two variables. Lastly, we see that the pointing of the polygons of the *worst* and *standard deviation* variables is far from their axes. These distributions of accuracies across the five variables demonstrate the discrepancies which exist in the performance of the top5 architectures. Clearly, the Top-1 architecture has the highest and best performance followed by the Top-2, then the Top-3, Top-4 and Top-5.

**Figure 10:**
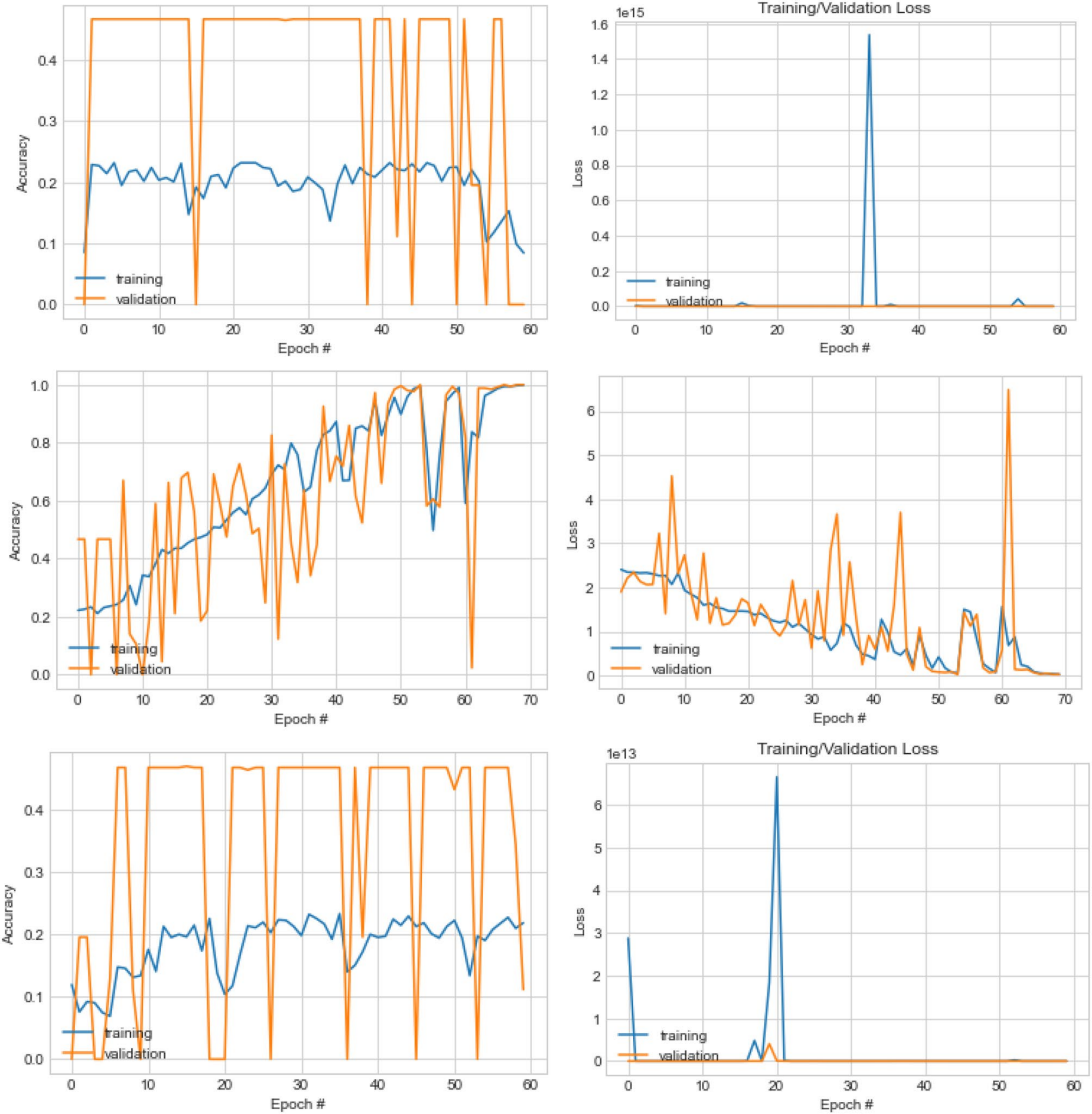
Plot of the accuracy and loss values for the training of the Top-1, 2, and 3 architectures respectively which were optimized using the EOSA-NAS model, showing their performances after sixty (60) training epoch.

Complete training of the top one (1) best performing architectures listed in Table 11 and illustrated in Fig. 10 showed that only the Top-1 and Top-2 demonstrated significant results. The two previous architectures overshadowed the outcome of those of Top-3 and Top-4. As a result, the Top-1 and Top-2 architectures were further evaluated beyond the 500 epochs of training. We found the Top-1 architecture converging well and learning the problem with impressive accuracy from the 60th epoch to the 100th epoch. Meanwhile, that of Top-2 architecture only began to show this stability later. This implies that the Top-1 architecture remains the best architecture that has learnt the classification problem well.

**Table 11:** Performance comparison for prediction of the four (4) best performing CNN architectures of the EOSA-NAS algorithm using AUC, precision, recall, sensitivity, specificity, accuracy and loss after full train for 60, 70 and 100 epochs.

Architectures	F1-score	Precision	Sensitivity	Specificity	Recall	Accuracy	Kappa
Top-4	0	0.0	–	–	0	0. 24	–
Top-2	0.1	0.1	0.1	0.1	0.1	0.1	0.1
Top-3	0	0	–	0	0.1	0. 25	0
Top-1	0.1	0.1	0.1	0.1	0.1	0.1	0.1

**Figure 11:**
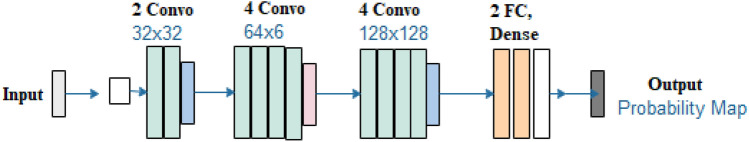
Neural network architecture of the Top-1 architecture optimized using EOSA-NAS model, which represents the overall best performing architecture after hundred (100) training epoch.

To fully evaluate and investigate the performance of the top five architectures, we experimented again with these architectures on larger datasets and allowed for training using a longer epoch. In Table 11, we see the performance of each of the architectures in terms of F1-score, precision, recall, sensitivity, specificity, accuracy and Kappa values after the full train. We applied the distributions of these variables to plot the boxplot of their corresponding values and found that an interesting distribution was seen for values in each distribution. Also, the result obtained from the Table showed that the architecture corresponding to the CNN model in Fig. 11 outputs the optimal performance with an accuracy of 0.1. This then reflects the most acceptable CNN configuration required to learn the problem of classification of digital histopathology images using deep learning.

**Figure 12:**
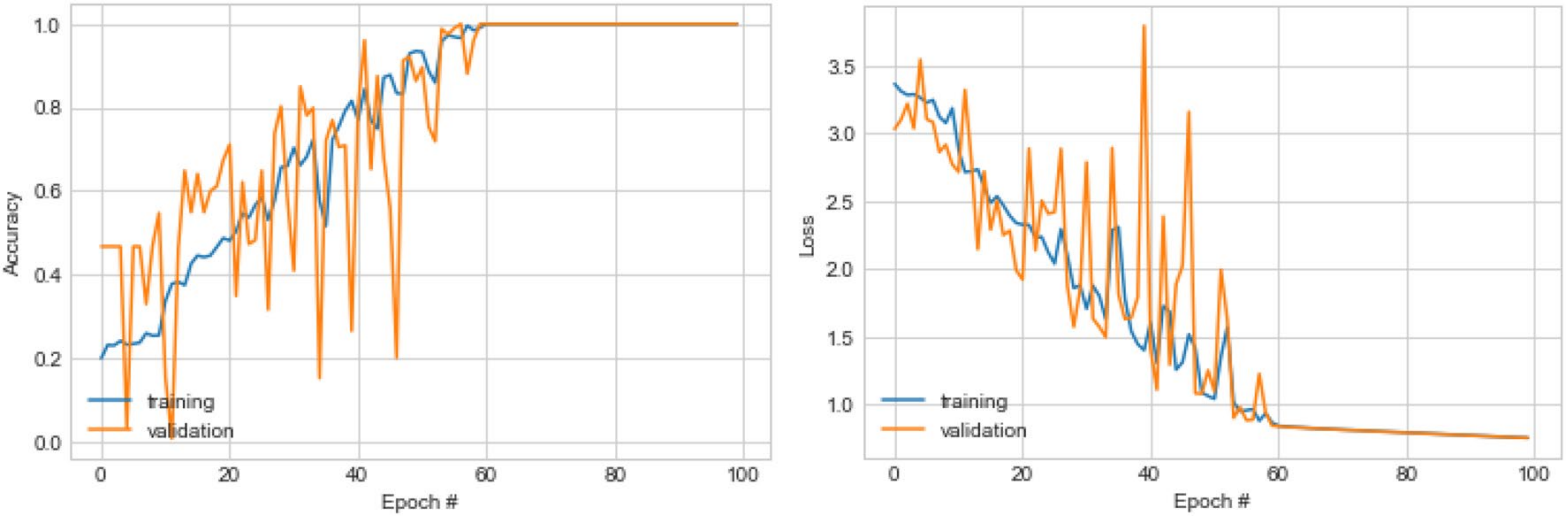
Plot of the accuracy and loss values for the training of the Top-1 architecture optimized using EOSA-NAS model, which represents the overall best performing architecture after hundred (100) training epoch.

Also, plotting the graph of the training phase of the Top-1 CNN model, we found that the loss function graph in Fig. 12 showed that the problem was learnt well as we see the loss values for those of training and validation overlapping as the training progressed. Similarly, the accuracy plot in the same figure demonstrates the evidence that the resulting CNN model is a candidate solution for consideration in future research on the application of deep learning to the classification of abnormalities in digital histopathology images.

**Figure 13:**
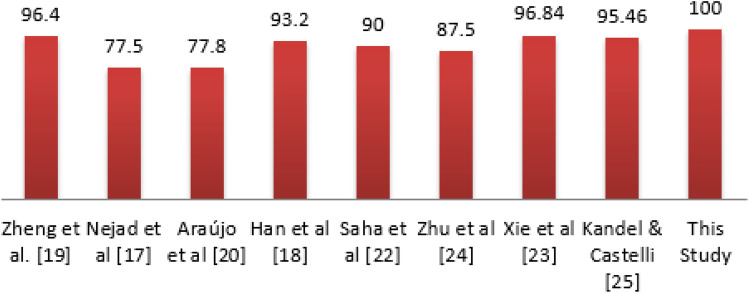
Comparison of the CNN architecture designed using EOSA-NAS model with state-of-the-art CNN architectures applied to the detection of breast cancer in histopathology images.

The result shown in Table 12 shows that most efforts in designing CNN models for histopathology image classification have all been approached using manual methods. Although the studies listed in the Table demonstrate some significant performance, the outcome of our experimentation confirms that automating the process is more beneficial. While the works of Zheng et al. ^19^ and Kandel and Castelli ^25^ compete with our method, we note that our method outperforms them. The graph in Fig. 13 shows a pictorial representation of the performance of all similar studies when compared with the outcome of this study.

**Table 12:** Comparison of NAS-based CNN design with state-of-the-art canonical CNN design approach for detection and classification of breast cancer using histopathology images.

References	Methods	Performance	Dataset
Zheng et al. ^19^	Nucleus-guided CNN	Accuracy 96.4%, Sensitivity 0.955, Specificity 0.964	Images from Motic (Xiamen) Medical Diagnostic Systems
Nejad et al. ^17^	CNN + Data augmentation	Detection rate 77.5%	BreakHis database
Araújo et al. ^20^	CNN + Support Vector Machine	Accuracies of 77.8%, sensitivity of 95.6%	Bioimaging 2015 breast histology classification challenge
Han et al. ^18^	Structured Deep Learning Model + Data augmentation	93.2% accuracy	BreakHis database
Saha et al. ^22^	Handcrafted features + CNN	92% precision, 88% recall and 90% *F*-score	MITOS-ATYPIA-14, ICPR-2012, and AMIDA-13 datasets
Zhu et al. ^24^	Squeeze-Excitation-Pruning (SEP) + CNN	Accuracy of 87.5*%*	BreaKHis and BACH dataset
Xie et al. ^23^	Inception_V3 and Inception_ResNet_V2	Accuracy 96.84%	BreaKHis
Kandel and Castelli ^25^	CNN	AUC of 95.46%	PatchCamelyon
Hägele et al. ^26^	CNN + explanation method	Improved AUC by 5%	BRCA
This study	EOSA-NAS CNN	Accuracy 100%	BreakHis and BACH databases

This study is focused on investigating the outcome of applying a NAS-based approach to the automation for the design of CNN architectures in the classification of breast histopathology images. The study aimed to address the difficulty in learning the problem associated with the domain. The outcome of the experimentation performed using EOSA-NAS based model for generating and optimising CNN architecture has proven very effective. This is based on the results obtained which have shown that applying the NAS approach to finding the best network configuration in detecting abnormalities in histopathology yields better performance. The accuracy obtained confirms that the application of the EOSA metaheuristic algorithm contributed to the overall performance of the NAS model. Meanwhile, this study has also shown that the proposed optimization algorithm, EOSA, competes well with similar state-of-the-art algorithms while showing superiority in the case of GA.

The EOSA metaheuristic algorithm was experimented with using fifteen (15) standard benchmark functions to demonstrate its viability and usefulness for solving optimization problems as in NAS model. Therefore, this study's finding confirms that automatic design for the CNN model in the classification task of histopathology images is more accurate than the manually designed models. Secondly, we showed that using the EOSA metaheuristic algorithm in a NAS-based model in optimizing purpose is also very positive. The approach in this study is in contrast to the widely adopted method for designing CNN architectures in learning the problem of detection of abnormalities in histopathology samples. Therefore, the proposed method offers a new order for the design of CNN architectures for this class of problem for the domain mentioned.

## Conclusion

This study demonstrates the importance of applying the NAS-based method to the challenge of designing CNN architectures. It further shows that applying the approach to learning abnormalities in histopathology images is of great benefit compared with the manual CNN design method. Moreover, the metaheuristic algorithm (EOSA) used to optimise the search strategy of the NAS model proves to be very relevant to tackling the problem. Although most studies that have applied deep learning to the task of detection and classification of breast histopathology images have shown some good performance, the findings of this study showed that using a NAS-based technique will improve detection and classification rate. The outstanding performance of the EOSA and NAS models hybridisation yielded a state-of-the-art CNN model that sufficiently learns the problem in the domain. The most interesting performance of the resulting CNN architecture is the values of the metrics: accuracy, sensitivity, specificity, precision, and recall, all leading to reduced classification error and reduced false-positive rates.

The outcome of this study demonstrates the evidence that the resulting CNN model remains a candidate solution for consideration in future research on the application of deep learning to the classification of abnormalities in digital histopathology images for the detection of breast cancer. The NAS strategy applied in this study and the resulting candidate architecture provides researchers with an understanding of network configuration suitable for using digital histopathology. However, the resulting top-5 and the best performing CNN architectures were trained to learn the classification problem of detecting abnormalities in histopathology images suggesting the presence of cancer. Hence, the performance may not measure up when applied to digital mammography.

In future, we recommend a comparative study investigating the performance of biology and swarm-based optimization algorithms in the use of search strategy for a NAS-based model. Considering the outstanding performance of the EOSA-NAS model proposed in this study, we recommend applying it to improve the search for configuring generative adversarial networks (GANs) for synthesizing histopathology images.
